# G Protein-Coupled Receptors in Prostate Cancer: Pathophysiological Implications and Potential for Innovative Therapeutics

**DOI:** 10.3390/ijms27209099

**Published:** 2026-10-14

**Authors:** Andre Sarmento-Cabral, Vicente Herrero-Aguayo, Prudencio Sáez-Martínez, Emilia Alors-Pérez, Antonio J. León-González, Juan L. López-Cánovas, Antonio J. Montero-Hidalgo, Manuel D. Gahete, Juan M. Jiménez-Vacas, Raúl M. Luque

**Affiliations:** 1 Department of Cell Biology, Physiology, and Immunology, University of Córdoba, 14014 Córdoba, Spain; 2 Maimonides Institute for Biomedical Research of Córdoba (IMIBIC), Hospital Universitario Reina Sofía (HURS), 14004 Córdoba, Spain; 3 Universidad Europea de Madrid, Faculty of Biomedical and Health Sciences, Department of Pharmacy and Nutrition, 28670 Madrid, Spain; 4 Department of Pharmacology, Faculty of Pharmacy, University of Seville, 41012 Seville, Spain; 5 Department of Medicine and Life Sciences, Universitat Pompeu Fabra, 08003 Barcelona, Spain; 6 Centre for Genomic Regulation (CRG), The Barcelona Institute of Science and Technology, 08003 Barcelona, Spain; 7 Centro de Investigación Biomédica en Red de Fisiopatología de la Obesidad y Nutrición (CIBERobn), 28019 Madrid, Spain; 8 The Institute of Cancer Research, London SM2 5GP, UK

**Keywords:** GPCR, prostate cancer, drugs, therapy, hormonal and inflammatory targets

## Abstract

Prostate cancer (PCa) is one of the leading causes of cancer-related death among men worldwide. Considerable efforts have been devoted to developing more effective therapeutic strategies for PCa, particularly for metastatic castration-resistant prostate cancer (mCRPC), wherein resistance to androgen-receptor pathway inhibition remains a major clinical challenge. G protein-coupled receptors (GPCRs), the largest family of cell surface signaling receptors, regulate numerous physiological and pathological processes and represent major therapeutic targets across multiple diseases, including cancer. In PCa, hormone- and inflammation-related GPCRs have emerged as key regulators of tumor initiation, progression, therapeutic resistance, and metastasis. Accordingly, understanding the contribution of GPCR signaling to PCa biology may reveal novel opportunities for therapeutic intervention, particularly in mCRPC. In this review, we summarize the current knowledge on the expression and function of GPCRs in PCa development, progression, and metastasis, highlighting their potential as therapeutic targets and discussing opportunities for drug development, combination therapies, and drug repurposing.

## 1. Introduction

**Figure 1:**
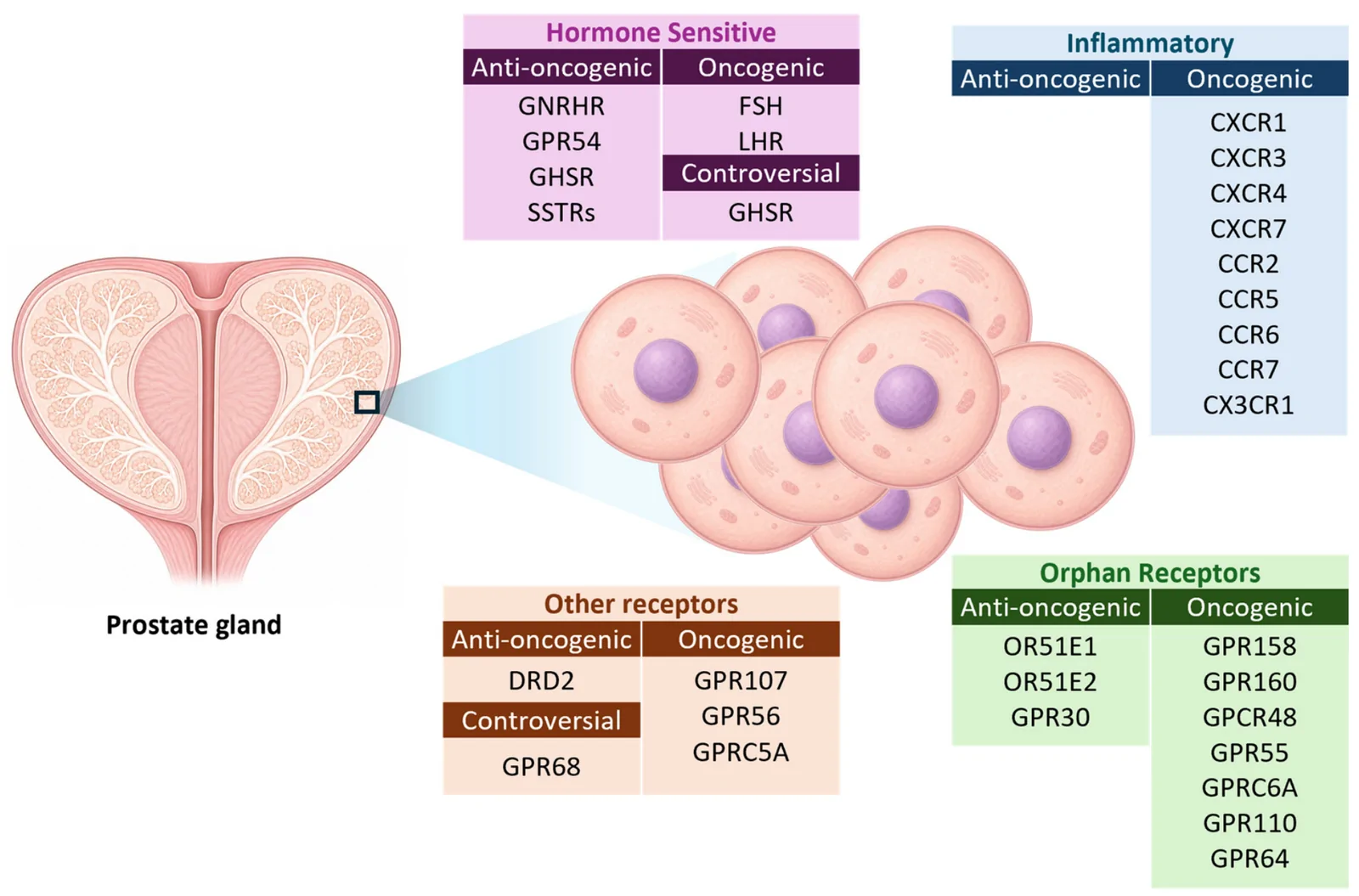
Representative summary of GPCRs effects over prostate gland cells considering its oncogenic/antioncogenic and controversial actions. Prostate and cells image generated with GPT-4o Image via Perplexity AI.

Prostate cancer (PCa) is the second most diagnosed cancer among men worldwide and represents one of the leading causes of cancer-related deaths [1]. As with many other cancer types, PCa is a heterogeneous pathology that presents complex molecular, hormonal and physiological characteristics, which converts its diagnosis, prognosis and treatment into a clinical and scientific challenge. Part of this challenge might be related to the fact that PCa is highly influenced by the hormonal environment [2]. In fact, the development and progression of PCa is tightly associated with androgen-receptor (AR) signaling [3]. Early-stage disease is typically androgen-dependent and initially responds to androgen deprivation therapy (ADT). However, under the selective pressure of ADT, advanced PCa eventually progresses to castration-resistant prostate cancer (CRPC), in which persistent AR signaling drives tumor growth despite castrate levels of circulating androgens [4]. CRPC represents the most lethal stage of the disease, and treatment options remain limited once resistance to currently available therapies develops. In addition to AR signaling, several hormones acting through GPCRs have been implicated in PCa development, progression, and aggressiveness (as illustrated in Figure 1). Of particular importance are hormonal systems from the central nervous system [e.g., follicle-stimulating hormone (FSH), luteinizing hormone (LH), gonadotropin-releasing hormone (GNRH), kisspeptin 1 (KISS1), ghrelin, somatostatin (SST), etc.] which impact PCa pathophysiology through the activation of their respective receptors (discussed in the following sections). Another key regulatory factor underlying PCa heterogeneity is chronic inflammation [5,6,7], since growing evidence suggests that chronic inflammation contributes to prostate carcinogenesis by promoting critical cellular processes, including immune dysregulation and remodeling of the tumor microenvironment [5,6,7]. Although the biological process of inflammatory response is correlated with infection, it may also be triggered by autoimmune diseases, allergies, and trauma of prostate tissue [8]. In this sense, inflammatory cells can express and release several cytokines capable of activating major signal cascades involved in cell growth (e.g., TNFα, CXCL8, IL6, etc.) [9]. In addition, inflammatory cell infiltration promotes tumor angiogenesis, DNA damage, cytoskeletal remodeling, and extracellular matrix degradation, thereby creating a tumor-supportive microenvironment [10].

In this context, GPCRs are the largest family of cell membrane-associated molecules mediating signal transmission in the mammalian genome [11]. Remarkably, these receptors are among the most popular drug targets, comprising around 36% of the current approved drugs [12]. Indeed, GPCR-related signaling cascades have been linked to critical cellular processes pivotal in tumorigenesis [13]. However, research into the involvement of GPCRs in cancer has been directed towards certain GPCR members only, with just a small number of FDA-approved GPCR drugs used for anticancer purposes. Thus, massive efforts are currently underway to identify effective targets for personalized treatment and to distinguish cancer-driver GPCRs from non-participant GPCRs. Considering the above, and the lack of effective treatments to palliate PCa, the study of the role of GPCRs in various biological processes, including cancer biology, might help identify promising drugs to manage PCa. Herein, we summarize the current knowledge on the presence and roles of multiple GPCRs in modulating cell signaling and PCa progression and metastasis, as well as the therapeutic potential of targeting these GPCRs (see Table 1).

## 2. Endocrine-Related GPCRs

As mentioned above, several mechanisms contribute to PCa progression, with persistent AR signaling remaining a central driver throughout disease evolution. In addition to the pivotal role of AR signaling, several hormone-responsive GPCRs have also been implicated in PCa development and progression (as illustrated in Figure 1), highlighting their potential as alternative therapeutic targets.

### 2.1. GnRHR

The gonadotropin-releasing hormone (GnRH) receptor (GnRHR), also known as the luteinizing-hormone releasing hormone (LHRH) receptor, is predominantly expressed in the cell membrane of pituitary gonadotrophs, wherein its stimulation by the hypothalamic hormone GnRH leads to the synthesis and secretion of gonadotropins (LH and FSH) [60]. This signaling is mediated by a cascade initiated after Gq/PLC activation [61], which involves PKC activation and Ca^2+^ efflux from endoplasmic reticulum, among other effects [62,63]. GnRHR has also been detected in different tumor tissues, including breast, prostate, ovarian, endometrial, pancreatic, and colorectal cancer, but acting through a different signaling mechanism [14,64]. In PCa, the GnRHR is coupled to a Gi protein, thereby reducing cancer cell proliferation and metastasis [14,15,64,65].

Due to the key role of this receptor in PCa progression, different therapeutic strategies have been designed to target GnRHR. Among them, GnRH agonists have been clinically used for decades in ADT. These agonists, such as goserelin, leuprolide, triptorelin, and histrelin, are analogs of GNRH more resistant to peptidases and, thus, with a prolonged half-life. Although GnRH agonists can induce an initial tumor flare due to transient activation of the hypothalamic–pituitary–gonadal (HPG) axis and increased testosterone levels, this effect is now largely prevented, at least in part, by concomitant treatment with antiandrogens (e.g., flutamide or bicalutamide) [66,67,68,69,70]. The sustained administration of high doses of these agonists desensitizes pituitary GnRHRs, blocking the HPG axis and finally reducing the testosterone circulating levels and significantly improving the PCa management compared to surgical castration [71,72]. Interestingly, it was recently shown that pharmacological relocalization of GnRHR to the cell membrane sensitizes PCa cells to radiotherapy in combination with leuprolide [73]. Additionally, it was uncovered that GV1001 (a fragment of the human telomerase reverse transcriptase catalytic subunit) can bind to GnRHR, reducing plasma testosterone levels, and inhibiting tumor growth and increasing apoptosis of LNCaP xenografts [16]. However, ADT with GnRH agonists is associated with an increased risk of osteoporosis, fatigue, and metabolic and cardiovascular diseases, among others [74,75,76,77,78,79,80,81,82]. Nevertheless, compared with GnRH agonists, degarelix has been associated with a lower incidence of major adverse cardiovascular events in some studies, although the available evidence remains conflicting [83].

A second strategy to target pituitary GnRHRs, but avoiding the flare phenomenon and reducing other undesirable side effects, consists of the treatment of GnRH antagonists [84]. These compounds are high-affinity, reversible and competitive inhibitors of GNRHR and induce a rapid reduction in gonadotropins after their administration [85,86]. Although the efficacy of first generations of GnRH antagonists was limited due to their low availability and the induction of allergic reactions [87], these undesirable characteristics have been improved in the third generation [88,89,90,91]. In addition to their effects though the HPG axis inhibition, GnRH analogs have also been shown to exert antitumor effects in vitro and in vivo through direct interaction with prostatic GnRHR [92]. As an example, the GnRH agonist goserelin and the antagonist degarelix exert antiproliferative effects (in vitro or in xenograft mouse models) on both androgen-dependent PCa cells such as LNCaP (goserelin and degarelix), and in the androgen-independent cell DU145 (goserelin) [89,90].

More recently, a third strategy targeting the prostatic expressed GnRHR has emerged based on receptor-targeted peptide-drug conjugates. Since GnRHR is overexpressed in PCa cells compared with normal prostate tissue, several GnRH-based peptide-drug conjugates have been developed to selectively deliver cytotoxic agents to tumor cells, thereby enhancing antitumor efficacy while reducing systemic toxicity [93]. Numerous studies have been conducted on a wide range of hybrid compounds that combine chemotherapeutic agents (e.g., D-melphalan, cyclopropane, doxorubicin, anthraquinones, methotrexate) with GnRH analogs that serve as drug delivery systems [94,95]. In this sense, one of the most advanced studies has reported the efficacy of a series of LHRH-doxorubicin hybrids, including AN-152 (also known as zoptarelin-doxorubicin, commercialized as AEZS-108), which have been suggested as a second- or third-line therapy for metastatic CRPC after the clinical benefit observed in a phase II clinical trial [96,97]. Other hybrid compounds include lytic peptides conjugated with GNRH and toxins (e.g., pokeweed antiviral protein, isolated from Phytolacca americana) [98,99].

### 2.2. FSHR and LHR

Although most of the PCa therapies focus on the HPG axis targeting GnRHR, other two key elements of this axis, FSH receptor (FSHR) and LH receptor (LHR), have raised growing interest as potential targets or diagnostic/prognostic biomarkers of PCa. Both FSHR and LHR belong to the rhodopsin-like GPCRs, are primarily expressed in gonads and exert a key role in reproduction of mammals [100].

Physiologically, activation of the FSHR in the granulosa cells of the ovary or the Sertoli cells of the testis leads to the canonical activation of the Gαs/cAMP/PKA cascade, triggering the induction of genes involved in steroidogenesis and spermatogenesis [100]. FSHR also activates other factors, including PKB (AKT), serum/glucocorticoid-regulated kinase (SGK), p38 MAPK, and the RAS/ERK1/2 signaling pathway [101,102,103]. Additionally, FSHR is expressed (at lower levels than in ovaries and testes) in several tissues such as endometrium, endothelial cells of the placenta, monocytes, and osteoclasts in physiological conditions [101,102,103]. Moreover, in different types of cancer (including ovarian and breast cancer), the activation of FSHR present in tumor epithelial cells induced cancer cell proliferation and migration [25,104,105], whereas the activation of vascular endothelial FSHR in tumor blood vessels was associated with the transendothelial transport of FSH and the angiogenesis process [106]. In PCa, several research groups reported the expression of FSH and FSHR by normal, benign hyperplasia and cancer tissue since the 1990s [23,107,108,109,110]. These investigations suggested a key role of the FSH/FSHR system in the progression of PCa, especially in the development of the castration-resistant status, as FSHR expression is significantly higher in PCa compared to benign prostatic hyperplasia (BPH) and normal prostate tissue [108], and exogenous FSH treatment has been associated with a proliferative effect in prostate cancer cell lines [23,24]. In fact, due to its higher expression in tumor tissues and vessels, FSHR has been proposed as biomarker for diagnostic purposes, including PCa [106,111].

Although the role of FSH/FSHR in PCa is still not fully understood, recent studies have also indicated that FSH promotes PCa xenograft tumor growth [25]. Conversely, suppression of FSHR signaling might serve as a potential therapeutic strategy against PCa [112]. Specifically, it has been reported that a synthetic DNA vaccine generating immune response against FSHR significantly delayed the tumor progression in mice that were inoculated with TRAMPC2 PCa cells expressing murine FSHR [112]. Additionally, different compounds, such as negative allosteric modulators of FSHR (ADX61623 and ADX68692) [113], were tested in PCa for their possible role targeting the FSH/FSHR axis, and have been described as potential nonsteroidal contraceptives, but their possible effects in PCa progression are still lacking [114].

In a similar way, LHR is also mainly expressed in the gonads, wherein it regulates the ovulation and the development of corpus luteum in females and the synthesis of testosterone in the Leydig cells of testes. These physiological actions are mainly mediated through the Gs/cAMP/PKA pathway, but other signaling pathways, such as Gq/PLC/Ca^2+^, have also been proposed [115]. Healthy prostate gland, BPH and PCa cells express LHR at mRNA and protein levels, as evidenced in murine models, human-derived samples, cell lines and xenografts by different techniques [108,116,117]. Importantly, it has been suggested that the LH/LHR signaling plays a key role in the development of castration resistance [118]. In fact, treatment with LH has been shown to induce an overexpression of steroidogenic genes and to increase testosterone levels and proliferation of PCa cell lines [26]. Moreover, certain genetic variants of LH and LHR, such as νLH and the LHR 312 G allele, have been associated with an increased risk of developing PCa and increased PCa-specific mortality [119].

Different groups have studied the effect of targeting the prostatic LHR as a potential treatment of CRPC. One of the therapeutic strategies consisted in a conjugate of a lytic peptide, such as Hecate, with a segment of the β chain of human LH (βLH)/chorionic gonadotropin (βCG), which induced cell death in a time- and concentration-dependent manner, with the magnitude of the effect correlating with LHR expression levels across different cell lines [120,121]. These results have been corroborated in vivo using xenograft models, with a significant decrease in tumor growth and metastasis observed in mice treated with this conjugate [122,123]. On the other hand, silencing LHR expression at the mRNA and protein levels using LHR-siRNA prevented LH-mediated induction of cell proliferation and androgen synthesis in different PCa cell lines [116]. The expression of key genes involved in PCa development (e.g., *AR*, *PSA*, *PKA*, *ERK1/2*, PI3K, *AKT2*, and *HER2*) was also downregulated in the LHR silenced cells [116]. Despite these promising results, further preclinical and clinical trials are still lacking to fully establish the basis of targeting prostate-specific LHR as an adjuvant therapy for PCa patients.

### 2.3. GPR54

*KISS1* encodes a precursor protein that is processed into several peptides, mainly kisspeptin-54. Kisspeptin-54 and other derived peptides (K-14, K-13, K-10) mediate their action by activating the specific receptor KISS1R [also known as GPR54 (G protein-coupled transmembrane receptor 54)], with the same affinity and efficacy [124], meaning that this system is expressed in several tumor types, including PCa [125,126]. To exert its functions, GPR54 couples primarily to Gq/11, activating PLCβ, IP3/DAG production, Ca^2+^ mobilization, PKC, and ERK1/2. In hypothalamic GnRH neurons, this pathway stimulates GnRH release and, secondarily, the secretion of LH and FSH that is essential for the onset of puberty, fertility, and the control of the hypothalamic–pituitary–gonadal axis [127,128].

In fact, kisspeptin was discovered due to its action in tumoral pathologies as a metastasis suppressor (called metastin before kisspeptin) [127]. Nowadays, the role of kisspeptins in cancer appears to be highly context-dependent, varying across tumor types, including pituitary adenomas [129], colorectal cancer [130] and pancreatic cancer [131], wherein they have been linked to distinct clinical outcomes and biological functions. Indeed, the role of kisspeptins and its receptor in PCa is not deeply explored. Analysis of PCa cell lines and prostatic tissue revealed the presence of both KISS1 and KISS1R [126]. Despite the synthesis and release of this peptide by various PCa cell lines, PCa patients do not exhibit elevated plasma kisspeptin levels [132]. In fact, the expression levels of Kisspeptin and KISS1R have been reported to decrease with the malignancy of analyzed tissues (with a lack of expression in metastasis) [133]. Furthermore, overexpression of KISS1 induced the suppression of metastasis progression and inhibition of the invasion process, in addition to the augmented chemosensitivity to paclitaxel [17], being part of these effects exerted via EIF2AK2 activation [17]. However, there are very few studies testing kisspeptin as a therapeutic treatment in PCa. Specifically, kisspeptin analogs showed a suppressive effect on testosterone levels in rats [134] with similar results in human PCa, even to castration levels [135]. Although these findings suggest that modulation of the kisspeptin pathway may influence androgen production, further studies are required to determine whether this approach has therapeutic potential as an alternative form of androgen deprivation. Nevertheless, treatment with kisspeptin or analogs must be carefully considered since it could cause testicular degeneration [136].

### 2.4. Ghrelin Receptor

The ghrelin receptor, originally known as growth hormone secretagogue receptor (GHSR), is a heterotrimeric G protein-coupled receptor [presenting two alternative splicing isoforms (GHSR1a and GHSR1b) [137]] that is involved in different biological processes, including food intake, hormonal secretion, and reproduction [138,139]. On the one hand, GHSR1a, a 366-amino acid receptor with 7 transmembrane domains, is activated by the acylated form of ghrelin hormone, a 28-amino acid peptide mainly secreted by the stomach [140], activating the Gq/PLC/PKC pathway [63], resulting in the release of calcium from internal storages. Although this is the main mechanism of action of this receptor, there are other signaling pathways involved in GHSR1a activation, such as AMPK, PI3K/AKT, and ERK1/2 pathway [63]. On the other hand, GHSR1b, a C-terminally truncated isoform of GHSR1a with 5 transmembrane domains, does not respond to ghrelin, and it has been suggested that GHSR1b traps GHSR1a into the endoplasmic reticulum, reducing its cell surface expression, acting as a dominant negative of GHSR1a signaling [141].

Although some reports have indicated that GHSRs are expressed in the prostate gland, their expression levels and roles in PCa cells remain controversial [142,143,144]. In fact, some studies revealed that exposing PC3 and LNCaP to ghrelin (10 mM)-induced activation of PI3K/AKT and ERK1/2 signaling pathways resulted in an increase in cell proliferation and the inhibition of apoptosis in PCa cells [22,145], while other studies showed an antiproliferative and proapoptotic effect of ghrelin in prostatic cells [18,19]. The apparently conflicting findings may be explained, at least in part, by the different concentrations of acylated ghrelin (the active form that activates GHSR1a) used across studies. It has been proposed that physiological concentrations of acylated ghrelin promote PCa aggressiveness, whereas supraphysiological concentrations may inhibit PCa cell growth. Nonetheless, the ghrelin axis is altered in PCa, where it may play an important and complex role and could serve as a source of new therapeutic targets for PCa treatment or as diagnostic biomarkers for early detection [18,144,146,147]. In fact, daily intraperitoneal administration of the ghrelin receptor antagonist [D-Lys3]-GHRP-6 in mice bearing PC-3 xenografts significantly reduced tumor volume and weight after two weeks of treatment. However, this antitumor effect was lost by day 18, suggesting that its therapeutic efficacy might be transient [20]. Finally, des-acyl ghrelin exerts both similar and opposite actions to those of the acylated form [142]. Particularly, ghrelin/des-acyl ghrelin inhibited DU-145 cell proliferation, displayed a biphasic effect in PC3 cells [i.e., low concentrations (10–100 pM) stimulated, whereas higher doses (1 nM to 1 µM) inhibited cell growth], and were ineffective in LNCaP cells [142]. This phenomenon, in addition to the fact that des-acyl ghrelin does not bind to GHSR1a/b, suggests the presence of undiscovered subtypes of GHSRs [142]. Moreover, it has been reported that a novel ghrelin splicing isoform, known as In1-ghrelin, is involved in PCa aggressiveness [144,148], although the receptor mediating these actions remains unknown, further supporting the idea that there are other GHSRs to be discovered.

### 2.5. Somatostatin Receptors

The somatostatin receptors (SSTRs) comprise a family of GPCRs consisting of 5 genes (*SSTR1*–*SSTR5*) located on chromosomes 14, 17, 22, 20, and 16, respectively. SSTRs respond to somatostatin-14/28 and couple to Gi/o proteins, reducing cAMP/PKA levels and modulating Ca^2+^, K^+^, MAPK/ERK, PI3K–AKT, and SHP phosphatases. The SSTRs mainly play an inhibitory role (e.g., inhibiting the secretion of insulin, glucagon, and growth hormone, among other hormones). In neuroendocrine neoplasms, it has been demonstrated that the expression of SSTR2 and SSTR5 supports the use of somatostatin analogs [149,150].

In the oncology field, the activation of these receptors has been associated with decreased proliferation and increased apoptosis across a wide variety of tumor cell types [151]. Particularly in PCa, it was shown that SST can reduce 22Rv1 and PC3 cells proliferation, migration, and colony formation [21]. Therefore, interest in SSTR research has been continually growing, and many SST analogs (SSAs), which show longer biological half-lives than SST, have been developed in recent years and are used in several clinical conditions [152]. Importantly, the actions and effectiveness of SSAs mainly depend on the presence of specific SSTRs on the membranes of tumor cells. Thus, octreotide and lanreotide preferentially bind to SSTR2 [153,154], while pasireotide binds to SSTR5 [155]. Importantly, the expression pattern of SSTRs is substantially different in the case of PCa, compared to other tumor-types. Specifically, the most expressed SSTR in PCa tissues is SSTR1 [156,157,158], which might play a constitutive pathophysiological role in this pathology. Indeed, it has been reported that SSTR1 overexpression (as well as a specific SSTR1 agonist BIM-23926) decreased cell proliferation in 22Rv1 cells, likely through the regulation of androgen-receptor (AR) activity and PI3K/AKT-CCND3 signaling-pathway [159]. Regarding SSTR2, immunohistochemistry results showed moderate to high levels of this receptor in only 13% of cases [160], which might explain the lack of effectiveness of octreotide in PCa patients [161]. However, considerable SSTR2 levels were detected in 50% of the analyzed neuroendocrine PCa in another study [160]. These findings, together with the observation that co-treatment with octreotide and docetaxel additively increased apoptosis in CRPC cells, suggest that octreotide could be explored as a potential treatment, either alone or in combination with ADT and/or chemotherapy, particularly in CRPC with neuroendocrine differentiation [162]. In the case of SSTR3 and 4, although they are present on the membrane of CRPC cells, their levels are lower than those detected in hormone-sensitive PCa and non-tumoral prostate samples [163]. Finally, positive immunohistochemical staining of SSTR5 in PCa samples has been reported [164,165]. In line with this, pasireotide and other SSTR5 analogs have been shown to reduce the proliferation rate of LNCaP cells, although these data should be interpreted with caution, as only a very limited number of samples were examined in this study [166]. Interestingly, SSTR5TMD4, an oncogenic truncated receptor generated by aberrant splicing of SSTR5 pre-mRNA [167], is overexpressed in PCa tissues and associated with aggressiveness parameters in PCa [168]. Mechanistically, this aberrant receptor blocks the canonical response to SSAs by promoting SSTR2 internalization [134], which may partly explain the limited clinical efficacy of SSTR5 analogs in PCa. Nevertheless, considering the different expression patterns of SSTRs in PCa, SSAs (preferentially those binding to SSTR1) may be used as a personalized treatment in PCa patients.

## 3. Inflammatory Receptors

As occurs in other types of cancer, PCa is strongly influenced by the inflammatory status of the gland [5,169]. Although many factors are involved in this chronic inflammation, chemokines (secreted proteins known for their roles in mediating immune cell trafficking and lymphoid tissue development) are one of the main drivers involved in this process [170,171] (as illustrated in Figure 1). Specifically, chemokines are the largest subfamily of cytokines and can be subdivided into four main classes depending on the location of the first two cysteine (C) residues in their protein sequences. Their actions are triggered by the ligand association with the respective GPCRs which may be classified in: CXC-chemokine receptors, CC-chemokine receptors, C-chemokine receptors (not described in PCa) and CX3C-chemokine receptors [172]. Therefore, taking into account the importance of inflammation in tumoral pathologies, these inflammatory receptors may have a key role in the response of the PCa cells to inflammatory microenvironment dysregulations [173,174].

### 3.1. CXC-Chemokine Receptors

This subfamily of chemokine receptors includes seven different receptors (CXCR1- CXCR7). CXCRs recognize CXC chemokines in a partially redundant manner. For example, CXCR1/2 respond to CXCL8/IL8 and other ELR+ chemokines; CXCR3 to CXCL9/10/11; CXCR4 to CXCL12; CXCR5 to CXCL13; and CXCR6 to CXCL16. CXCRs primarily couple to Gi/o proteins, activating PI3K–AKT, PLCβ–Ca^2+^, RAS–RAF–MEK–ERK, RAC/RHO, and FAK, while CXCR7 (currently named ACKR3) signals mainly via β-arrestin and regulates CXCL12 availability. Collectively, these receptors control chemotaxis, adhesion, extravasation, lymphoid organization, angiogenesis, and tumor infiltration, with the CXCL12–CXCR4 axis being particularly relevant in metastasis and cellular niches [175,176].

CXCR1 and CXCR2 are expressed on PCa cells, including metastatic lesions [177] and have been associated with increased tumor progression, angiogenesis and also, incidence of lymph node metastasis [177,178,179]. Different studies have shown that CXCR1 activation by CXCL8 promotes mitogenic response in PCa cells [27,28]. In fact, this function as a mitogenic activator seems to be more pronounced in CRPC cell lines [180]. Although several drugs target both receptors, only G31P, a CXCR1/2 antagonist, inhibits the growth of human PCa cell lines in immunodeficient mice [181]. Moreover, CXCL12-CXCR4/CXCR7 might be considered one of the most important inflammatory axes in cancer (including PCa) [182] since these receptors and their ligand, CXCL12, have been associated with an increased cell proliferation, differentiation, epithelial–mesenchymal transition, and metastasis of PCa [30,31,32,33]. Interestingly, resveratrol and AMD3100 have emerged as the most promising inhibitors of CXCR4, reducing invasion and metastatic features in different in vivo and in vitro models [183,184].

Likewise, some CXCRs (specifically the CXCR2 and CXCR3) have been shown to have opposite effects in PCa, particularly regarding angiogenesis. For instance, tumors in TRAMP/CXCR3^−/−^ mice were detectable much earlier and had much increased angiogenesis than those found in control TRAMP/CXCR3^+/+^ mice [29]. Additionally, the transformation of prostatic epithelial cells of the TRAMP/CXCR3^−/−^ mice tumors resulted in an increased production of pro-angiogenic chemokines and a decrease in angiostatic chemokines [29]. In fact, TRAMP/CXCR2^−/−^ mice presented smaller tumors than control TRAMP/CXCR2^+/+^ mice and reduced angiogenesis [29]. Interestingly, other studies have shown that two different CXCR3 mRNA isoforms are expressed in different tumor types including prostate cells, being the levels of CXCR3A increased in PCa while CXCR3b were under-expressed [185]. Importantly, the dysregulation in the balance between both isoforms has been associated with metastatic process [186]. Likewise, CXCR5/6/7 have also been associated with progression and metastasis of PCa. Indeed, IL30 plays an important role in regulating PCa stem-like cell behavior and metastatic potential though the CXCR5/CXCL13 axis (lymph nodes and bone marrow metastasis) [35]. Also, CXCR7, in addition to CXCR4, has been described as a relevant factor in cell growth, survival and adhesion of PCa cells [36]. Of note, in CRPC patients, CXCR7 overexpression stimulated by AR antagonists was associated with better response to enzalutamide [187]. Nevertheless, another study concluded that patients with high expression levels of CXCR7 presented a higher probability of PCa recurrence after radical prostatectomy [188]. Despite the relevance of these receptors in PCa, a limited number of drugs have been tested in PCa. For instance, it has been reported that andrographolide inhibits PCa by reducing CXCR3 and CXCR7 expression [189], and that a CXCR7 agonist (CCX771) in combination with Enzalutamide suppresses cell growth and angiogenic signaling in CRPC models [190]. Furthermore, it was recently published that balizafortide, a CXCR4 inhibitor, enhances the docetaxel antitumoral effects in a PCa bone metastatic mouse model [34], while a clinical trial with a CXCR2 inhibitor (AZD5069) plus enzalutamide in patients with mCRPC that are resistant to AR signaling inhibitors showed that a CXCR2 blockade inhibited senescence-associated, tumor-elicited myeloid inflammation, with this blockade reversing AR signaling inhibitors resistance to confer durable antitumor activity in a subset of patients with mCRPC [191].

### 3.2. C-C-Chemokine Receptors

The second widest subfamily of chemokine receptors (CCRs) is composed of 10 different receptors (CCR1–CCR10). CCRs recognize CC chemokines, including CCL2–CCR2, CCL3/CCL4/CCL5–CCR5, CCL17/CCL22–CCR4, CCL19/CCL21–CCR7, CCL25–CCR9, and CCL27/CCL28–CCR10. Predominant coupling to Gαi/o induces a decrease in cAMP and the activation of PLCβ, PI3K–AKT, ERK/MAPK, Rho-GTPases, as well as actin cytoskeleton remodeling. Additionally, CCR signaling is further regulated by GRKs, β-arrestins, desensitization, and internalization. These receptors determine the recruitment and positioning of monocytes, lymphocytes, dendritic cells, and eosinophils, thereby playing a role in inflammation, mucosal immunity, autoimmunity, infection, and the tumor microenvironment [175,192].

In PCa, only some CCRs (CCR2, CCR5, CCR6 and CCR7) have been characterized. Specifically, CCR2 has been reported as a key regulator for the development of BPH, and for PCa progression [193]. In fact, the CCL2/CCR2 axis was implicated in the resistance to cabazitaxel through the inhibition of caspase-3 and poly (ADP-ribose) polymerase [37], and contributed to cell migration of cabazitaxel resistant PCa cells [38]. Moreover, an association between CCR5 and increased PCa cell proliferation has been reported [39]. Then, other studies have corroborated the oncogenic role of the CCL5/CCR5 axis in PCa [194,195]. Notably, some studies have shown that blocking this receptor may be a therapeutic approach for treating PCa, even reducing metastasis to bone [196,197]. Furthermore, an oncogenic role for CCR6 has been suggested, acting as a migration inducer following AR suppression in PCa cell models [40]. Finally, CCR7 has also been described as an oncogene, especially in the development of PCa metastasis. Indeed, CCR7 regulates the Notch1 pathway by increasing activation of the MAPK and NF-κB signaling pathways [41]. In addition, it was suggested that TNFα induces CCR7 expression and that the CCL21/CCR7 axis might increase the metastatic potential of PCa cells during lymph node metastasis [42].

### 3.3. CX3C-Chemokine Receptor

CX3CR1, or GPR13, is the sole member of this receptor subfamily of receptors sensitive to the specific ligand CX3CL1/fractalkine, a chemokine that exists in both transmembrane (adhesive) and soluble (chemotactic) forms. It predominantly couples to Gi proteins and activates PI3K–AKT, PLCβ/Ca^2+^, ERK/MAPK, Src/FAK, and, depending on the contexts, JAK2–STAT3. Its activation results in the promotion of adhesion, migration, survival, and angiogenesis. The CX3CL1–CX3CR1 axis regulates the trafficking of monocytes, Natural Killer cells, cytotoxic lymphocytes, and microglia, and has been implicated in atherosclerosis, neuroinflammation, metastasis, and antitumor immunity [198,199].

The association between PCa and CX3CR1 has been reported, which promotes cell adhesion, migration, and survival mechanisms during the metastasis process [43]. Additional studies have shown that this receptor is implicated in metastasis, highlighting bone marrow [200] and spinal metastasis [201], being in the last case through the regulation of EGFR, SRC and FAK pathways [202]. Finally, more recent studies have also suggested that CX3CR1 signaling orchestrates malignant progression of PCa through luminal progenitor-macrophage crosstalk [203], and that this receptor could serve as a biomarker associated with pathological tumor staging, playing a role in predicting PCa prognosis [204].

Altogether, although further studies are warranted in this field, the inhibition of these chemokine receptors might be a promising therapeutic avenue for PCa, particularly for the treatment of metastatic PCa.

## 4. Orphan Receptors

Some GPCRs are called orphan G-protein-coupled receptors because their ligands are either unknown or may not exist. It is known that some orphan G protein-coupled receptors regulate many physiological processes, and alterations in their function can lead to various diseases [205,206,207,208,209]. In this sense, some of these receptors are dysregulated in PCa, wherein they play an important role in the development and progression of this pathology [210] (as illustrated in Figure 1). Therefore, the study of the pathophysiological role of these receptors is necessary, since it could provide novel tools for diagnostic, prognosis, and the therapy of PCa and other endocrine-related cancers [211].

In this line, the Olfactory Receptor Family 51 Subfamily E Member 1 and 2 (OR51E1 and OR51E2) are seven-transmembrane domain olfactory receptors expressed outside the olfactory epithelium and prostate gland. OR51E1 responds to nonanoic acid, whereas OR51E2 can be activated by β-ionone, acetate, and propionate. Both receptors can signal via the modulation of cAMP and intracellular Ca^2+^, as well as the activation of kinases such as Src/Pyk2, ERK1/2, or p38 MAPK, although the response depends on the ligand and the cell model [44,212,213]. Both receptors have been found to be overexpressed in PCa samples compared to non-tumoral samples [44]. In fact, OR51E1 and OR51E2 [also known as PSGRs (Prostate-specific GPCRs) due to their association with PCa] have been proposed as potential diagnostic tools and new therapeutic targets [212,214,215,216]. Indeed, the overexpression of these receptors in LNCaP cells induced apoptosis and decreased cell proliferation [44]. Moreover, a recent report described that activation of OR51E1 reduced PCa cell survival, an effect that was abolished in OR51E1 knockout cells [217]. Interestingly, this study identified a previously unrecognized GPCR signaling axis that suppresses PCa cell survival by demonstrating that sphingosine-1-phosphate receptor 1 (S1PR1) is a key modulator of OR51E1 function in PCa cells. Indeed, co-expression of OR51E1 and S1PR1, but not OR51E1 alone, was significantly associated with a longer progression-free interval in patients with PCa, particularly those with stage II disease [217].

Likewise, GPR158 has been shown to induce PCa. GPR158 forms complexes with RGS7/Gβ5 proteins and regulates Gi/o signaling, reducing cAMP levels and modulating ion channels. It is expressed in the brain and prostate, wherein it has been linked to synaptic plasticity, stress responses, and depression, as well as to PCa progression and resistance to androgen deprivation therapy [218]. Moreover, it has been reported that GPR158 induces proliferation in PCa cells independently of AR function and that increased GPR158 expression in tumors was associated with reduced disease-free survival [45].

Another receptor highly expressed in PCa is GPR160 which is associated with PCa progression via ERK and CREB [219], and its silencing induced apoptosis and cell cycle arrest [46]. Another oncogenic orphan G protein-coupled receptor found in PCa is GPR55. The strongest pharmacological evidence points to lysophosphatidylinositol (LPI) as a candidate endogenous agonist and primarily couples to G12/13 and activates RhoA–ROCK, PLCβ, oscillatory Ca^2+^ release, ERK1/2, p38, and transcription factors, but it may also exhibit coupling to Gq or Gi, depending on the context. GPR55 is involved in neuronal excitability, nociception, inflammation, metabolism, bone remodeling, and cell proliferation/migration, with potential implications for cancer [220,221]. In this line, its role is believed to be the stimulation of cell proliferation and anchorage-independent growth in PCa cells [47]. Furthermore, GPRC6A is considered another orphan receptor, which is activated by basic amino acids, osteocalcin, and testosterone, although the exact physiological contribution of some of these ligands remains uncertain. It preferentially couples to Gq/11, activating PLCβ, IP_3_, Ca^2+^, PKC, ERK1/2, and the PI3K–AKT (context-dependent) pathway. It has been linked to energy and mineral homeostasis, endocrine secretion, testicular function, and metabolic adaptation [222]. GPRC6A was reported to participate in PCa regulating non-genomic effects of androgens [48]. Finally, GPR64 (currently named ADGRG2), an adhesion receptor highly expressed in the epididymis and male reproductive tract, signals via Gs–cAMP/PKA and, depending on the context, via Gq/11–PLC–Ca^2+^, ERK/MAPK, and RhoA. GPR64 is essential for epididymal function, luminal fluid reabsorption, and sperm maturation, and pathogenic variants are associated with congenital bilateral absence of the vas deferens and male infertility [223,224]. This receptor was reported to be overexpressed in PCa suggesting that it could exert oncogenic roles [225]; however, there is no evidence of in vitro or in vivo functional effects in PCa models.

## 5. Other GPCRs

In addition to the receptor families mentioned above, there are other GPCRs that have been reported to play important roles on PCa. Particularly, dopamine receptors (DRs) are activated by dopamine and preferentially couple to Gs/Golf, increasing cAMP/PKA levels and modulating DARPP-32, CREB, and ERK. More specifically, DRD2 couple to Gi/o, reducing cAMP, inhibiting Ca^2+^ channels, activating GIRK channels, and regulating the β-arrestin–AKT–GSK3β pathway, wherein these pathways have been linked to control movement, reward, cognition, motivated behavior, and neuroendocrinology [226]. DRD2 may also control different parameters of aggressiveness such as cell death, proliferation, invasion, and migration [227] (as illustrated in Figure 1). Indeed, the pathophysiological role of DRD2 has been proposed in a wide variety of tumor pathologies wherein this receptor is overexpressed and associated with altered tumoral progression [228]. Recent studies in PCa have demonstrated that thioridazine treatment (an antagonist of DRD2) can induce differentiation of cancer stem cell-like cells by inhibiting phosphorylation of AMPK [49]. Additionally, pimozide (a DRD2 inhibitor) reduces PCa cells proliferation [50]. Conversely, stimulation of DRD2 by a bromocriptine agonist induces cell-cycle arrest via inhibition of the expression of c-MYC, E2F1, survivin, and AR, and increases the expression of several cell-cycle suppressors (p53, p21, p27) [51]. Therefore, these studies suggest that DRD2 could have an important role in the differentiation and progression of PCa and be a good candidate to develop PCa therapies.

As previously mentioned, certain components of the SST system are dysregulated in PCa, wherein these alterations seem to influence its development and progression. In this regard, the functional roles of neuronostatin (encoded by the somatostatin gene [229]) and its receptor GPR107 (that may activate PI3K/AKT, β-arrestin/ERK/NF-κB [230,231]) have been recently revealed in PCa [52]. Specifically, GPR107 is overexpressed in PCa and associated with key clinical parameters in advanced stages of PCa. Furthermore, silencing this receptor inhibited proliferation and migration in androgen-independent PCa models. In fact, treating PCa cells with neuronostatin also promoted the inhibition of proliferation and migration of androgen-independent PCa cells, and stimulated the reduction in GPR107 receptor expression [52].

Another GPCR recently implicated in PCa aggressiveness is GPR56 (ADGRG1) as its activation is required for proper androgen signaling in PCa cells [53]. GPR56 is an adhesion receptor that interacts with collagen III, transglutaminase 2, and tetraspanin CD81, among other context-dependent regulators, and it primarily activates the G12/13–RhoA–ROCK pathway and adhesion signaling, being essential for cortical organization and neuronal migration, hematopoietic stem cell generation, immunoregulation, muscle development, and reproduction [232].

Additionally, GPRC5A was associated with the formation of PCa metastases. GPRC5A is a retinoic acid-inducible class C GPCR whose signaling function is highly dependent on the cellular context. Available evidence linked GPRC5A to the modulation of cAMP/PKA/CREB, EGFR/c-Src/STAT3, gp130/JAK/STAT3, FAK/Src, and NF-κB axes, as well as with pathways associated with cytoskeletal reorganization, migration, survival, and tumor progression [233]. Specifically, PC3 cells with absence of GPRC5A expression by CRISPR/Cas9 KO presented reduced cell proliferation in vitro and in vivo and failed to develop bone metastases in xenograft mice models [54].

GPR68, a proton-sensing GPCR that responds to extracellular acidity and signals primarily via the Gq/11–PLCβ–IP3/DAG–Ca2+–PKC pathway, involving the activation of Rho, ERK1/2, p38, and cAMP (depending on the context), plays a role in acidosis sensing, immune function, inflammation, bone homeostasis, and adaptation to the acidic tumor microenvironment, with effects that can be either pro- or anti-tumorigenic depending on the specific cell and tissue types [234]. In the PCa context, GPR68 knockout promoted tumorigenicity reduction in TRAMP-C2 cells in GPR68 KO mice [55], but other studies have suggested a controversial role of GPR68 in PCa since its overexpression in PC3 cells reduced cell migration, invasion, and metastasis formation [56].

Furthermore, GPCR48, known as LGR4 and activated by norrin, R-spondins (RSPOs), and the ligand of the receptor activator of nuclear factor kappa-B (RANKL) [235], can promote tumorigenesis in normal prostate, via PI3K/AKT, an important oncogenic signaling pathway accentuated in CRPC, being thus considered as a potential biomarker of the progression of this disease [57]. In contrast, GPR30 (currently named GPER1 and activated by estrogens [236]) exerts the opposite effect, reducing cell growth in vitro and in vivo [58], via Gs, cAMP/PKA, Ca^2+^, Src, ERK/MAPK, PI3K-AKT [237,238]. Finally, GPR110 (currently named ADGRF1, activated by synaptamide [239], and overexpressed in PCa [59]) exerts oncogenic roles through the activation of PKA/CREB [240].

## 6. Interactions Between GPCR and Androgen-Receptor Signaling

Resistance to androgen-receptor signaling inhibitors (ARSIs) in mCRPC is characterized by the reactivation of androgen signaling, increased lineage plasticity, and marked transcriptional heterogeneity. In this sense, GPCRs may promote resistance to AR-pathway inhibitors by enabling signaling networks that are not fully dependent on ligand-bound AR activity. In particular, GPCR-mediated activation of β-arrestin, MAPK/ERK, PI3K/AKT, Src, PKC, and Rho-family pathways can sustain proliferation, survival, motility, and cellular plasticity during pharmacologic AR suppression [241,242]. In fact, these intracellular pathways may activate or stabilize AR signaling. As an example, MAPK driven phosphorylation of YB1, via either activated ERK or RSKs, may increase AR transcriptionally [243]. In the same line, activation of the PI3K/AKT pathway may promote AR signaling through two complementary mechanisms: AKT-mediated phosphorylation of AR, which modulates AR stability and activity in a cell-context-dependent manner, and phosphorylation-dependent nuclear exclusion/inactivation of FOXO1, a transcriptional repressor of both full-length AR and AR-V7 [244,245,246,247].

Another way of interaction with AR signaling is AR splice-variant induction. Long-term ADT increases NF-κB signaling, and this may increase AR variants such as AR-V7, which remain active without the ligand-binding domain targeted by enzalutamide and abiraterone [241]. Finally, GPCR signaling may also promote lineage plasticity. Under AR pathway inhibition, prostate tumors may undergo neuroendocrine shift, an AR-indifferent state linked to resistance, and GPCRs signaling has been linked to therapy-induced neuroendocrine PCa models [242,248,249,250]. When the other way around, it was recently shown that resistant tumors exhibited amplification of a downstream AR enhancer alongside SSTR1 suppression. Accordingly, reduced SSTR1 expression was associated with lower clinical efficacy of subsequent ARSIs. It was also shown that SSTR1 exerted an antiproliferative effect in 22Rv1 cells, and treatment with pasireotide inhibited cell proliferation. Collectively, it was proposed that SSTR1 loss could be a mechanism associated with ARSI resistance and its pharmacological activation may arise as a potential therapeutic strategy for ARSI-resistant mCRPC [251].

## 7. Clinical Applications of GPCR Targeting in PCa Treatment

Currently, clinical applications for targeting GPCRs in PCa are essentially directed to GnRHR. GnRH agonists induce pituitary desensitization, while antagonists directly block the receptor, suppressing LH and testosterone as the basis of androgen ADT [66,67,68,69,70,71,72], and are commonly combined with AR pathway inhibitors and chemotherapy [252]. Among non-endocrine receptors, a CXCR2 antagonist, AZD5069, was tested combined with enzalutamide in the ACE trial (NCT03177187), showing signs of activity alongside a reduction in neutrophils and immunosuppressive myeloid cells [191], whereas navarixin plus pembrolizumab demonstrated limited activity in mCRPC in the phase II trial (NCT03473925) [253]. In the same line, SX-682, a dual CXCR1/CXCR2 inhibitor, is being evaluated in mCRPC in combination with enzalutamide in the SYNERGY-201 trial (NCT06228053), with apalutamide in the ASpiRE trial (NCT07002320), and with docetaxel in a trial that includes an advanced PCa cohort (NCT07667400).

Furthermore, SSTRs have been investigated without achieving standard-of-care status. Octreotide LAR was evaluated in androgen-independent PCa (NCT00510224), showing no PSA decline despite a reduction in IGF1 [254], while pasireotide, a SSTR1/2/3/5 agonist (NCT01468532), was found to be feasible and safe when combined with docetaxel/prednisone in mCRPC [255]. More recently, SSTR2 has been adopted as a target for imaging and radioligand therapy in metastatic neuroendocrine prostate carcinoma in the NCT06379217 trial. Regarding all other receptors referred to in this review, we could not find information about therapeutic trials.

Most of the referred clinical trials are focused on combined therapies, rather than single agent therapies. The combination may enhance the activity of standard therapies in advanced PCa. Other studies have focused on the targeting combination of GPCRs, yet without testing as clinical trials. In CRPC models, CXCR7 is upregulated after AR-pathway inhibition and promotes enzalutamide resistance through β-arrestin 2-dependent MAPK/ERK signaling. Accordingly, CXCR7 blockade enhances the antitumor effects of enzalutamide, reducing tumoral behavior parameters [256,257]. GPCR targeting may also increase taxane efficacy. Inhibition of G protein βγ signaling increased paclitaxel activity in PCa cells and xenograft tumors, while CXCR4 inhibition with balixafortide enhanced docetaxel antitumoral activity in a mouse model of PCa bone metastasis [190,258]. In contrast, there is a lack of evidence about the combinations of GPCR-targeted therapies with radiotherapy or immunotherapy in the context of PCa.

Despite the growing evidence supporting GPCRs as therapeutic targets in PCa, different translational challenges to clinical practice should be considered. Intra- and inter-tumoral heterogeneity in GPCR expression may restrict therapeutic efficacy to specific patient subgroups and favor the selection of receptor-negative or receptor-low tumor cell populations [259,260]. Moreover, receptor desensitization and internalization may reduce the magnitude and duration of therapeutic responses [261], while signaling plasticity and the activation of alternative pathways may lead to treatment resistance [260]. Furthermore, the ligands specificity and the structural similarity among related GPCR subtypes [262,263], added to the dose dependency effect, compromise the development of specific and selective drugs. On the other side, the expression of many GPCRs in normal tissues increases the risk of off-target and on-target/off-tumor toxicity [264]. Finally, characterization of the GPCR mutations and expression levels variation in PCa [260,265] may be crucial for the correct patient’s stratification, with the final objective of selecting the most effective treatment, and for the development of robust biomarkers.

## 8. Final Remarks and Conclusions

Collectively, the evidence discussed in this review highlights GPCRs as central regulators of PCa biology, influencing tumor growth, metastasis, treatment resistance and tumor–microenvironment interactions (summarized in Table 1). While GnRH-targeted therapies are already established in clinical practice, increasing evidence supports the therapeutic potential of additional GPCR families, including FSHR, chemokine receptors and SSTRs. However, some receptors have shown a dual effect that might be dependent on both the dose and tested model. Particularly, depending on the cell lines tested, low concentrations of ghrelin/desacyl ghrelin stimulated cell growth, whereas higher doses inhibited cell growth. Another example is GPR68, which also exhibits context-dependent effects, with its knockout reducing tumorigenicity in TRAMP-C2 cells and its overexpression suppressing it in PC3 cells.

Furthermore, most of these targets remain at the preclinical stage, and future studies should prioritize functional validation, patient stratification, biomarker development and rational combination strategies with current standard-of-care therapies. In fact, despite preclinical evidence supporting the involvement of several GPCRs in PCa, to the best of our knowledge, the GnRH receptor remains the only GPCR currently targeted in clinical practice to reduce systemic androgen signaling. In addition, a small number of clinical trials have evaluated agents targeting CXCR1/CXCR2 and SSTRs, either in combination with immunotherapy or androgen-receptor inhibitors. Unfortunately, these studies have not yielded significant clinical benefits.

Additionally, GPCRs act as modulators of tumor, stromal, endothelial, and immune cells within the tumor microenvironment. In this sense, cytokines and inflammation are particularly important for prostate tumor microenvironment alterations [266]. In the stroma, receptors such as CXCR4 activate JAK-STAT and PI3K-AKT pathways in fibroblasts, promoting matrix remodeling and inflammation [267]. In the immune compartment, chemoreceptors like CXCR2 recruit neutrophils, macrophages, and Myeloid-derived suppressor cell that generate immunosuppression [268,269]. In this line, CCL2–CCR2 may also favor monocyte recruitment and polarization toward M2 tumor-associated macrophages, contributing to invasion, metastasis, and therapeutic resistance [173,270]. Finally, endothelial GPCRs drive neovascularization, and receptors such as CCR7 and CCR10 may impact extravasation and metastatic seeding via Rho GTPases and matrix metalloproteinases [173,271].

Thus, studies focusing on a GPCR multi-omic approach in cancer tissues and cell/preclinical models (genomics, transcriptomics, proteomics, interactomics, etc.), as well as on GPCR-related molecular mechanisms associated with tumor progression and metastasis should be seriously considered. In the same line, determining whether a GPCR exerts a pro- or anti-tumor function in PCa depending on the ligand concentration is essential for making a rational decision as to whether its activation by agonists or its inhibition by antagonists might offer the greatest therapeutic benefit.

These approaches could explore and characterize functionally important endogenous GPCRs associated with health and disease taking into account not only the tumor cellular behavior, but also the interaction with the tumor microenvironment. Such advances could ultimately facilitate the incorporation of GPCR-targeted approaches into precision medicine (e.g., with improved diagnostic, prognostic, and treatment tools) for patients with advanced PCa.

## Figures and Tables

**Table 1:** Direct effect of ligand-receptor on prostate cancer cell models.

Type	Receptor	Ligand	Effect(s)	Model(s)	References
Hormone sensitive	GNRHR	GNRH	Reduce cell proliferation and metastasis. Increase apoptosis	LNCaP, DU145, PC3 (in vitro)	[14,15,16]
	GPR54	KISS1	Suppress cell migration and invasion processes	PC3 (in vitro)	[17]
	GHSR	Ghrelin	Antiproliferative and proapoptotic effects	DU145, PC3 (in vitro) PC3 (in vivo)	[18,19,20]
	SSTR1,2,5	SST	Reduce proliferation, migration, and colony formation	LNCaP, 22Rv1, PC3 (in vitro)	[21]
	GHSR	Ghrelin	Increase cell proliferation	PC3, LNCaP (in vitro)	[22]
	FSHR	FSH	Increase proliferation and tumor growth	PC3, DU145 (in vitro and in vivo)	[23,24,25]
	LHR	LH	Increase proliferation	LNCaP (in vitro)	[26]
Inflammatory	CXCR1	CXCL8	Promote mitogenic response	PC3, DU145 (in vitro)	[27,28]
	CXCR2	CXCL8 CXCL1 CXCL2	Reduction in the expression leads to tumor size reduction	TRAMP/CXCR2^−/−^ mice	[29]
	CXCR3	CXCL9 CXCL10 CXCL11	May serve as tumor suppressor	TRAMP/CXCR3^−/−^ mice	[29]
	CXCR4 CXCR7	CXCL12	Promote cell proliferation, differentiation, epithelial–mesenchymal transition, and metastasis	PC3, DU145, 22Rv1 (in vitro), HiMyc mice	[30,31,32,33]
	CXCR4	CXCL12	Inhibition sensitizes to docetaxel	PC3 (in vivo)	[34]
	CXCR5	CXCL13	Increase metastatic potential	PIN-SC (in vitro), TRAMP mice (in vivo)	[35]
	CXCR7	CXCL12 CXCL11	Promote cellular proliferation and resistance to apoptosis	LNCaP (in vivo)	[36]
	CCR2	CCL2	Promote resistance to cabazitaxel and stimulates cell migration	DU145 (in vivo)	[37,38]
	CCR5	CCL5	Induce cell proliferation	DU145, LNCaP (in vitro)	[39]
	CCR6	CCL20	Induce cell migration	LNCaP, C4-2B (in vitro)	[40]
	CCR7	CCL21	Favor cell migration and metastasis formation	PC3, LNCaP, DU145 (in vitro)	[41,42]
	CX3CR1	CX3CL1	Promote adhesion, migration, survival and metastasis formation	PC3, LNCaP, DU145 (in vitro)	[43]
Orphan	OR51E1 OR51E2		Apoptosis inducers and proliferation inhibitors	LNCaP (in vitro)	[44]
	GPR158		Induce cell proliferation	LNCaP, C4-2B, PC3, DU145 (in vitro)	[45]
	GPR160		Apoptosis suppressor	PC3, LNCaP (in vitro). PC3 (in vivo)	[46]
	GPR55		Stimulation of cell proliferation	PC3 (in vitro)	[47]
	GPRC6A		Support testosterone signaling	22Rv1 (in vitro), GPRC6A^−/−^ mice (in vivo)	[48]
Other	DRD2	DA	Inhibition induces differentiation of cancer stem cell-like cells	Stem cell-like cells derived from PC3; DU145, LNCaP) (in vitro)	[49,50]
	DRD2	DA	Stimulation induces cell-cycle arrest	C4-2 (in vitro)	[51]
	GPR107	NST	Both silencing and stimulation promote cell proliferation	22Rv1, PC3 (in vitro)	[52]
	GPR56	Collagen III and TG2	Required for androgen signaling and induce cell growth	LNCaP, PC3 (in vitro)	[53]
	GPRC5A	TG2 heparin	Important for cell proliferation and metastasis formation.	PC3 (in vitro and in vivo)	[54]
	GPR68	pH sensor	Promote tumorigenesis.	*Ogr1*^−/−^ mice	[55]
			Cell proliferation, migration and tumor formation suppression	PC3 (in vivo and in vitro)	[56]
	GPCR48 (LGR4)	RSPOs, norrin, RANKL	Promotes tumorigenesis	PC3, DU145 (in vitro and in vivo)	[57]
	GPR30 (GPER1)	Estrogens	Reduces cell growth	PC3 (in vivo)	[58]
	GPR110 (ADGRF1)	synaptamide	Proposed as oncogenic receptor	LNCaP, PC3 (in vitro)	[59]

## Data Availability

No new data were created or analyzed in this study. Data sharing is not applicable to this article.

## Abbreviations

The following abbreviations are used in this manuscript:

ADT	Androgen Deprivation Therapy
AR	Androgen Receptor
ACKR3	Atypical Chemokine Receptor 3
ADGRF1	Adhesion G protein-coupled receptor F1 (GPR110)
BPH	Benign Prostatic Hyperplasia
CCL-#	Chemokine (C-C motif) Ligand
CCR-#	C-C chemokine receptor
CRPC	Castration-Resistant Prostate Cancer
CX3CL1	C-X3-C motif chemokine ligand 1
CX3CR1	C-X3-C motif chemokine receptor 1
CXCL-#	C-X-C motif chemokine ligand-#
CXCR-#	CXC chemokine receptor-#
DA	Dopamine
DRD2	Dopamine Receptor D2
FSH	Follicle-Stimulating Hormone
FSHR	FSH receptor
GPER1	G protein-coupled estrogen receptor 1 (GPR30)
GHSR	Growth Hormone Secretagogue Receptor
GHSR1a	Growth Hormone Secretagogue Receptor isoform 1a
GHSR1b	Growth Hormone Secretagogue Receptor isoform 1b
GNRH	Gonadotropin-Releasing Hormone
GNRHR	GNRH Receptor
GPCR	G Protein-Coupled Receptor
GPCR48	Leucine-rich Repeat Containing G Protein-coupled Receptor 4
GPCRs	G protein-coupled receptors
GPR30	G Protein-coupled Estrogen Receptor 1
GPR54	Kisspeptin Receptor
GPR55	G Protein-coupled Receptor 55
GPR56	Adhesion G Protein-coupled Receptor G1
GPR68	G protein-coupled receptor 68
GPR107	G protein-coupled receptor 107
GPR110	Adhesion G protein-coupled Receptor F1
GPR158	G Protein-coupled Receptor 158
GPR160	G protein-coupled Receptor 160
GPRC5A	G protein-coupled Receptor Class C group 5 Member A
GPRC6A	G protein-coupled Receptor Class C Group 6 Member A
HPG	Hypothalamic-Pituitary-Gonadal
IL6	Interleukin-6
KISS1	Kisspeptin 1
KISS1R	Kisspeptin Receptor
LGR4	Leucine-rich repeat-containing G-protein coupled receptor 4 (GPCR48)
LH	Luteinizing Hormone
LHR	LH receptor
LHRH	Luteinizing-Hormone Releasing Hormone
mCRPC	Metastatic Castration-Resistant Prostate Cancer
NST	Neuronostatin
OR51E-#	Olfactory Receptor Family 51 Subfamily E-#
PCa	Prostate Cancer
RANKL	Receptor Activator of Nuclear Factor Kappa-B Ligand
RSPOs	R-spondins
S1PR1	Sphingosine-1-Phosphate Receptor 1
SST	Somatostatin
SSTR	Somatostatin Receptor
TNFa	Tumor Necrosis Factor alpha

## References

[1] Filho A.M., Laversanne M., Ferlay J., Colombet M., Piñeros M., Znaor A., Parkin D.M., Soerjomataram I., Bray F. (2025). The GLOBOCAN 2022 cancer estimates: Data sources, methods, and a snapshot of the cancer burden worldwide. Int. J. Cancer.

[2] Sarmento-Cabral A., Fuentes-Fayos A.C., Ordoñez F.M., León-González A.J., Martínez-Fuentes A.J., Gahete M.D., Luque R.M. (2025). From pituitary cells to prostate gland in health and disease: Direct and indirect endocrine connections. Rev. Endocr. Metab. Disord..

[3] Hou Z., Huang S., Li Z. (2021). Androgens in prostate cancer: A tale that never ends. Cancer Lett..

[4] Bournakis E., Efstathiou E., Varkaris A., Wen S., Chrisofos M., Deliveliotis C., Alamanis C., Anastasiou I., Constantinides C., Bamias A. (2011). Time to castration resistance is an independent predictor of castration-resistant prostate cancer survival. Anticancer Res..

[5] Pérez-Gómez J.M., Montero-Hidalgo A.J., Fuentes-Fayos A.C., Sarmento-Cabral A., Guzmán-Ruiz R., Malagón M.M., Herrera-Martínez A.D., Gahete M.D., Luque R.M. (2023). Exploring the role of the inflammasomes on prostate cancer: Interplay with obesity. Rev. Endocr. Metab. Disord..

[6] Inamura K. (2018). Prostatic cancers: Understanding their molecular pathology and the 2016 WHO classification. Oncotarget.

[7] Nguyen D.P., Li J., Tewari A.K. (2014). Inflammation and prostate cancer: The role of interleukin 6 (IL-6). BJU Int..

[8] De Nunzio C., Kramer G., Marberger M., Montironi R., Nelson W., Schröder F., Sciarra A., Tubaro A. (2011). The Controversial Relationship Between Benign Prostatic Hyperplasia and Prostate Cancer: The Role of Inflammation. Eur. Urol..

[9] Zhao H., Wu L., Yan G., Chen Y., Zhou M., Wu Y., Li Y. (2021). Inflammation and tumor progression: Signaling pathways and targeted intervention. Signal Transduct. Target. Ther..

[10] Xie Y., Liu F., Wu Y., Zhu Y., Jiang Y., Wu Q., Dong Z., Liu K. (2025). Inflammation in cancer: Therapeutic opportunities from new insights. Mol. Cancer.

[11] Liu S., Anderson P.J., Rajagopal S., Lefkowitz R.J., Rockman H.A. (2024). G Protein-Coupled Receptors: A Century of Research and Discovery. Circ. Res..

[12] Lorente J.S., Sokolov A.V., Ferguson G., Schiöth H.B., Hauser A.S., Gloriam D.E. (2025). GPCR drug discovery: New agents, targets and indications. Nat. Rev. Drug Discov..

[13] Dorsam R.T., Gutkind J.S. (2007). G-protein-coupled receptors and cancer. Nat. Rev. Cancer.

[14] Limonta P., Montagnani Marelli M., Mai S., Motta M., Martini L., Moretti R.M. (2012). GnRH receptors in cancer: From cell biology to novel targeted therapeutic strategies. Endocr. Rev..

[15] Limonta P., Dondi D., Moretti R.M., Maggi R., Motta M. (1992). Antiproliferative effects of luteinizing hormone-releasing hormone agonists on the human prostatic cancer cell line LNCaP. J. Clin. Endocrinol. Metab..

[16] Kim J.W., Yadav D.K., Kim S.J., Lee M.Y., Park J.M., Kim B.S., Kim M.H., Park H.G., Kang K.W. (2019). Anti-cancer effect of GV1001 for prostate cancer: Function as a ligand of GnRHR. Endocr.-Relat. Cancer.

[17] Kim T.H., Cho S.G. (2017). Kisspeptin inhibits cancer growth and metastasis via activation of EIF2AK2. Mol. Med. Rep..

[18] Lawnicka H., Mełeń-Mucha G., Motylewska E., Mucha S., Stępień H. (2012). Modulation of ghrelin axis influences the growth of colonic and prostatic cancer cells in vitro. Pharmacol. Rep. PR.

[19] Díaz-Lezama N., Hernández-Elvira M., Sandoval A., Monroy A., Felix R., Monjaraz E. (2010). Ghrelin inhibits proliferation and increases T-type Ca^2+^ channel expression in PC-3 human prostate carcinoma cells. Biochem. Biophys. Res. Commun..

[20] Maugham M.L., Seim I., Thomas P.B., Crisp G.J., Shah E.T., Herington A.C., Gregory L.S., Nelson C.C., Jeffery P.L., Chopin L.K. (2019). Limited short-term effects on human prostate cancer xenograft growth and epidermal growth factor receptor gene expression by the ghrelin receptor antagonist [D-Lys(3)]-GHRP-6. Endocrine.

[21] Sáez-Martínez P., Porcel-Pastrana F., Pérez-Gómez J.M., Pedraza-Arévalo S., Gómez-Gómez E., Jiménez-Vacas J.M., Gahete M.D., Luque R.M. (2022). Somatostatin, Cortistatin and Their Receptors Exert Antitumor Actions in Androgen-Independent Prostate Cancer Cells: Critical Role of Endogenous Cortistatin. Int. J. Mol. Sci..

[22] Yeh A.H., Jeffery P.L., Duncan R.P., Herington A.C., Chopin L.K. (2005). Ghrelin and a novel preproghrelin isoform are highly expressed in prostate cancer and ghrelin activates mitogen-activated protein kinase in prostate cancer. Clin. Cancer Res. An. Off. J. Am. Assoc. Cancer Res..

[23] Ben-Josef E., Yang S.Y., Ji T.H., Bidart J.M., Garde S.V., Chopra D.P., Porter A.T., Tang D.G. (1999). Hormone-refractory prostate cancer cells express functional follicle-stimulating hormone receptor (FSHR). J. Urol..

[24] Dizeyi N., Trzybulska D., Al-Jebari Y., Huhtaniemi I., Lundberg Giwercman Y. (2019). Cell-based evidence regarding the role of FSH in prostate cancer. Urol. Oncol..

[25] Oduwole O.O., Poliandri A., Okolo A., Rawson P., Doroszko M., Chrusciel M., Rahman N.A., Serrano de Almeida G., Bevan C.L., Koechling W. (2021). Follicle-stimulating hormone promotes growth of human prostate cancer cell line-derived tumor xenografts. FASEB J. Off. Publ. Fed. Am. Soc. Exp. Biol..

[26] Pinski J., Xiong S., Wang Q., Stanczyk F., Hawes D., Liu S.V. (2011). Effect of luteinizing hormone on the steroidogenic pathway in prostate cancer. Prostate.

[27] MacManus C.F., Pettigrew J., Seaton A., Wilson C., Maxwell P.J., Berlingeri S., Purcell C., McGurk M., Johnston P.G., Waugh D.J. (2007). Interleukin-8 signaling promotes translational regulation of cyclin D in androgen-independent prostate cancer cells. Mol. Cancer Res. MCR.

[28] Shamaladevi N., Lyn D.A., Escudero D.O., Lokeshwar B.L. (2009). CXC Receptor-1 Silencing Inhibits Androgen-Independent Prostate Cancer. Cancer Res..

[29] Shen H., Schuster R., Lu B., Waltz S.E., Lentsch A.B. (2006). Critical and opposing roles of the chemokine receptors CXCR2 and CXCR3 in prostate tumor growth. Prostate.

[30] Taichman R.S., Cooper C., Keller E.T., Pienta K.J., Taichman N.S., McCauley L.K. (2002). Use of the stromal cell-derived factor-1/CXCR4 pathway in prostate cancer metastasis to bone. Cancer Res..

[31] Mochizuki H., Matsubara A., Teishima J., Mutaguchi K., Yasumoto H., Dahiya R., Usui T., Kamiya K. (2004). Interaction of ligand-receptor system between stromal-cell-derived factor-1 and CXC chemokine receptor 4 in human prostate cancer: A possible predictor of metastasis. Biochem. Biophys. Res. Commun..

[32] Ahn S., Saha A., Clark R., Kolonin M.G., DiGiovanni J. (2022). CXCR4 and CXCR7 signaling promotes tumor progression and obesity-associated epithelial-mesenchymal transition in prostate cancer cells. Oncogene.

[33] Gritsina G., Fong K.W., Lu X., Lin Z., Xie W., Agarwal S., Lin D., Schiltz G.E., Beltran H., Corey E. (2023). Chemokine receptor CXCR7 activates Aurora Kinase A and promotes neuroendocrine prostate cancer growth. J. Clin. Investig..

[34] Robinson T., Escara-Wilke J., Dai J., Zimmermann J., Keller E.T. (2023). A CXCR4 inhibitor (balixafortide) enhances docetaxel-mediated antitumor activity in a murine model of prostate cancer bone metastasis. Prostate.

[35] Sorrentino C., Ciummo S.L., Cipollone G., Caputo S., Bellone M., Di Carlo E. (2018). Interleukin-30/IL27p28 Shapes Prostate Cancer Stem-like Cell Behavior and Is Critical for Tumor Onset and Metastasization. Cancer Res..

[36] Begley L.A., MacDonald J.W., Day M.L., Macoska J.A. (2007). CXCL12 activates a robust transcriptional response in human prostate epithelial cells. J. Biol. Chem..

[37] Natsagdorj A., Izumi K., Hiratsuka K., Machioka K., Iwamoto H., Naito R., Makino T., Kadomoto S., Shigehara K., Kadono Y. (2019). CCL2 induces resistance to the antiproliferative effect of cabazitaxel in prostate cancer cells. Cancer Sci..

[38] Natsagdorj A., Izumi K., Hiratsuka K., Naito R., Kadomoto S., Iwamoto H., Yaegashi H., Shigehara K., Nakata H., Mizokami A. (2023). The CCL2-CCR2 Axis Contributes to Migration of Cabazitaxel-resistant Prostate Cancer Cells. Anticancer Res..

[39] Vaday G.G., Peehl D.M., Kadam P.A., Lawrence D.M. (2006). Expression of CCL5 (RANTES) and CCR5 in prostate cancer. Prostate.

[40] Kano H., Izumi K., Hiratsuka K., Toriumi R., Nakagawa R., Aoyama S., Kamijima T., Shimada T., Naito R., Kadomoto S. (2023). Suppression of androgen receptor signaling induces prostate cancer migration via activation of the CCL20-CCR6 axis. Cancer Sci..

[41] Du R., Tang G., Tang Z., Kuang Y. (2019). Ectopic expression of CC chemokine receptor 7 promotes prostate cancer cells metastasis via Notch1 signaling. J. Cell. Biochem..

[42] Maolake A., Izumi K., Natsagdorj A., Iwamoto H., Kadomoto S., Makino T., Naito R., Shigehara K., Kadono Y., Hiratsuka K. (2018). Tumor necrosis factor-α induces prostate cancer cell migration in lymphatic metastasis through CCR7 upregulation. Cancer Sci..

[43] Shulby S.A., Dolloff N.G., Stearns M.E., Meucci O., Fatatis A. (2004). CX3CR1-fractalkine expression regulates cellular mechanisms involved in adhesion, migration, and survival of human prostate cancer cells. Cancer Res..

[44] Pronin A., Slepak V. (2021). Ectopically expressed olfactory receptors OR51E1 and OR51E2 suppress proliferation and promote cell death in a prostate cancer cell line. J. Biol. Chem..

[45] Patel N., Itakura T., Jeong S., Liao C.P., Roy-Burman P., Zandi E., Groshen S., Pinski J., Coetzee G.A., Gross M.E. (2015). Expression and functional role of orphan receptor GPR158 in prostate cancer growth and progression. PLoS ONE.

[46] Zhou C., Dai X., Chen Y., Shen Y., Lei S., Xiao T., Bartfai T., Ding J., Wang M.W. (2016). G protein-coupled receptor GPR160 is associated with apoptosis and cell cycle arrest of prostate cancer cells. Oncotarget.

[47] Piñeiro R., Maffucci T., Falasca M. (2011). The putative cannabinoid receptor GPR55 defines a novel autocrine loop in cancer cell proliferation. Oncogene.

[48] Pi M., Parrill A.L., Quarles L.D. (2010). GPRC6A mediates the non-genomic effects of steroids. J. Biol. Chem..

[49] Lee S.I., Roney M.S.I., Park J.H., Baek J.Y., Park J., Kim S.K., Park S.K. (2019). Dopamine receptor antagonists induce differentiation of PC-3 human prostate cancer cell-derived cancer stem cell-like cells. Prostate.

[50] Zhou W., Chen M.K., Yu H.T., Zhong Z.H., Cai N., Chen G.Z., Zhang P., Chen J.J. (2016). The antipsychotic drug pimozide inhibits cell growth in prostate cancer through suppression of STAT3 activation. Int. J. Oncol..

[51] Yang Y., Mamouni K., Li X., Chen Y., Kavuri S., Du Y., Fu H., Kucuk O., Wu D. (2018). Repositioning Dopamine D2 Receptor Agonist Bromocriptine to Enhance Docetaxel Chemotherapy and Treat Bone Metastatic Prostate Cancer. Mol. Cancer Ther..

[52] Sáez-Martínez P., Jiménez-Vacas J.M., León-González A.J., Herrero-Aguayo V., Montero Hidalgo A.J., Gómez-Gómez E., Sánchez-Sánchez R., Requena-Tapia M.J., Castaño J.P., Gahete M.D. (2020). Unleashing the Diagnostic, Prognostic and Therapeutic Potential of the Neuronostatin/GPR107 System in Prostate Cancer. J. Clin. Med..

[53] Singh J.P., Dagar M., Dagar G., Kumar S., Rawal S., Sharma R.D., Tyagi R.K., Bagchi G. (2020). Activation of GPR56, a novel adhesion GPCR, is necessary for nuclear androgen receptor signaling in prostate cells. PLoS ONE.

[54] Sawada Y., Kikugawa T., Iio H., Sakakibara I., Yoshida S., Ikedo A., Yanagihara Y., Saeki N., Győrffy B., Kishida T. (2020). GPRC5A facilitates cell proliferation through cell cycle regulation and correlates with bone metastasis in prostate cancer. Int. J. Cancer.

[55] Yan L., Singh L.S., Zhang L., Xu Y. (2014). Role of OGR1 in myeloid-derived cells in prostate cancer. Oncogene.

[56] Singh L.S., Berk M., Oates R., Zhao Z., Tan H., Jiang Y., Zhou A., Kirmani K., Steinmetz R., Lindner D. (2007). Ovarian Cancer G Protein–Coupled Receptor 1, a New Metastasis Suppressor Gene in Prostate Cancer. JNCI J. Natl. Cancer Inst..

[57] Liang F., Yue J., Wang J., Zhang L., Fan R., Zhang H., Zhang Q. (2015). GPCR48/LGR4 promotes tumorigenesis of prostate cancer via PI3K/Akt signaling pathway. Med. Oncol..

[58] Chan Q.K., Lam H.M., Ng C.F., Lee A.Y., Chan E.S., Ng H.K., Ho S.M., Lau K.M. (2010). Activation of GPR30 inhibits the growth of prostate cancer cells through sustained activation of Erk1/2, c-jun/c-fos-dependent upregulation of p21, and induction of G(2) cell-cycle arrest. Cell Death Differ..

[59] Lum A.M., Wang B.B., Beck-Engeser G.B., Li L., Channa N., Wabl M. (2010). Orphan receptor GPR110, an oncogene overexpressed in lung and prostate cancer. BMC Cancer.

[60] Seeburg P.H., Mason A.J., Stewart T.A., Nikolics K. (1987). The mammalian GnRH gene and its pivotal role in reproduction. Recent Prog. Horm. Res..

[61] Grosse R., Schmid A., Schöneberg T., Herrlich A., Muhn P., Schultz G., Gudermann T. (2000). Gonadotropin-releasing hormone receptor initiates multiple signaling pathways by exclusively coupling to G(q/11) proteins. J. Biol. Chem..

[62] Naor Z. (2009). Signaling by G-protein-coupled receptor (GPCR): Studies on the GnRH receptor. Front. Neuroendocrinol..

[63] Yin Y., Li Y., Zhang W. (2014). The growth hormone secretagogue receptor: Its intracellular signaling and regulation. Int. J. Mol. Sci..

[64] Liu Y.F., Fu S.Q., Yan Y.C., Gong B.B., Xie W.J., Yang X.R., Sun T., Ma M. (2021). Progress in Clinical Research on Gonadotropin-Releasing Hormone Receptor Antagonists for the Treatment of Prostate Cancer. Drug Des. Dev. Ther..

[65] Limonta P., Moretti R.M., Montagnani Marelli M., Motta M. (2003). The biology of gonadotropin hormone-releasing hormone: Role in the control of tumor growth and progression in humans. Front. Neuroendocrinol..

[66] Kang Y.J., Kim K.H., Lee K.S. (2014). Efficacy of Bicalutamide 150-mg Monotherapy Compared with Combined Androgen Blockade in Patients with Locally Advanced Prostate Cancer. Korean J. Urol..

[67] Labrie F. (2014). GnRH agonists and the rapidly increasing use of combined androgen blockade in prostate cancer. Endocr.-Relat. Cancer.

[68] Akaza H., Hinotsu S., Usami M., Arai Y., Kanetake H., Naito S., Hirao Y. (2009). Combined androgen blockade with bicalutamide for advanced prostate cancer: Long-term follow-up of a phase 3, double-blind, randomized study for survival. Cancer.

[69] Zhao J., Chen J., Sun G., Shen P., Zeng H. (2024). Luteinizing hormone-releasing hormone receptor agonists and antagonists in prostate cancer: Effects on long-term survival and combined therapy with next-generation hormonal agents. Cancer Biol. Med..

[70] Iversen P., Damber J.E., Malmberg A., Persson B.E., Klotz L. (2016). Degarelix monotherapy compared with luteinizing hormone-releasing hormone (LHRH) agonists plus anti-androgen flare protection in advanced prostate cancer: An analysis of two randomized controlled trials. Ther. Adv. Urol..

[71] Østergren P.B., Kistorp C., Fode M., Bennedbaek F.N., Faber J., Sønksen J. (2019). Metabolic consequences of gonadotropin-releasing hormone agonists vs orchiectomy: A randomized clinical study. BJU Int..

[72] Østergren P.B., Kistorp C., Fode M., Henderson J., Bennedbæk F.N., Faber J., Sønksen J. (2017). Luteinizing Hormone-Releasing Hormone Agonists are Superior to Subcapsular Orchiectomy in Lowering Testosterone Levels of Men with Prostate Cancer: Results from a Randomized Clinical Trial. J. Urol..

[73] Sánchez C., Mercado A., Contreras H.R., Carvajal V.F., Salgado A., Huidobro C., Castellón E.A. (2021). Membrane translocation and activation of GnRH receptor sensitize prostate cancer cells to radiation. Int. J. Radiat. Biol..

[74] Crawford E.D., Schally A.V., Pinthus J.H., Block N.L., Rick F.G., Garnick M.B., Eckel R.H., Keane T.E., Shore N.D., Dahdal D.N. (2017). The potential role of follicle-stimulating hormone in the cardiovascular, metabolic, skeletal, and cognitive effects associated with androgen deprivation therapy. Urol. Oncol..

[75] Melloni C., Roe M.T. (2020). Androgen deprivation therapy and cardiovascular disease. Urol. Oncol..

[76] Greenspan S.L., Coates P., Sereika S.M., Nelson J.B., Trump D.L., Resnick N.M. (2005). Bone loss after initiation of androgen deprivation therapy in patients with prostate cancer. J. Clin. Endocrinol. Metab..

[77] Haque R., UlcickasYood M., Xu X., Cassidy-Bushrow A.E., Tsai H.T., Keating N.L., Van Den Eeden S.K., Potosky A.L. (2017). Cardiovascular disease risk and androgen deprivation therapy in patients with localised prostate cancer: A prospective cohort study. Br. J. Cancer.

[78] Hsueh J.Y., Gallagher L., Koh M.J., Shah S., Danner M., Zwart A., Ayoob M., Kumar D., Leger P., Dawson N.A. (2024). The impact of neoadjuvant relugolix on multi-dimensional patient-reported fatigue. Front. Oncol..

[79] Bosco C., Bosnyak Z., Malmberg A., Adolfsson J., Keating N.L., Van Hemelrijck M. (2015). Quantifying observational evidence for risk of fatal and nonfatal cardiovascular disease following androgen deprivation therapy for prostate cancer: A meta-analysis. Eur. Urol..

[80] Keating N.L., O’Malley A.J., Freedland S.J., Smith M.R. (2010). Diabetes and cardiovascular disease during androgen deprivation therapy: Observational study of veterans with prostate cancer. J. Natl. Cancer Inst..

[81] Wei J.T., Gross M., Jaffe C.A., Gravlin K., Lahaie M., Faerber G.J., Cooney K.A. (1999). Androgen deprivation therapy for prostate cancer results in significant loss of bone density. Urology.

[82] Muniyan S., Xi L., Datta K., Das A., Teply B.A., Batra S.K., Kukreja R.C. (2020). Cardiovascular risks and toxicity—The Achilles heel of androgen deprivation therapy in prostate cancer patients. Biochim. Biophys. Acta. Rev. Cancer.

[83] de Moraes F.C.A., Sano V.K.T., Dantas C.R., Hoffmeister N., Kelly F.A., Burbano R.M.R. (2024). A systematic review and meta-analysis of cardiovascular disease risk with degarelix and GnRH agonists in prostate cancer. Clin. Transl. Oncol..

[84] Margel D., Peer A., Ber Y., Shavit-Grievink L., Tabachnik T., Sela S., Witberg G., Baniel J., Kedar D., Duivenvoorden W.C.M. (2019). Cardiovascular Morbidity in a Randomized Trial Comparing GnRH Agonist and GnRH Antagonist among Patients with Advanced Prostate Cancer and Preexisting Cardiovascular Disease. J. Urol..

[85] Hopmans S.N., Duivenvoorden W.C., Werstuck G.H., Klotz L., Pinthus J.H. (2014). GnRH antagonist associates with less adiposity and reduced characteristics of metabolic syndrome and atherosclerosis compared with orchiectomy and GnRH agonist in a preclinical mouse model. Urol. Oncol..

[86] Kittai A.S., Blank J., Graff J.N. (2018). Gonadotropin-Releasing Hormone Antagonists in Prostate Cancer. Oncology.

[87] Engel J.B., Schultze-Mosgau A., Diedrich K. (2005). Five years’ clinical use of GnRH antagonists: Evaluation of safety and allergic potential. Reprod. Biomed. Online.

[88] Dellis A., Papatsoris A. (2017). Therapeutic outcomes of the LHRH antagonists. Expert Rev. Pharmacoecon. Outcomes Res..

[89] Tomar P., Jain N., Agarwal G.S., Dixit V.K. (2012). Goserelin loaded nanoparticles inhibit growth and induce apoptosis in human prostate cancer cell lines. Drug Deliv. Transl. Res..

[90] Sakai M., Martinez-Arguelles D.B., Patterson N.H., Chaurand P., Papadopoulos V. (2015). In search of the molecular mechanisms mediating the inhibitory effect of the GnRH antagonist degarelix on human prostate cell growth. PLoS ONE.

[91] Zou F., Wang Y., Yu D., Liu C., Lu J., Zhao M., Ma M., Wang W., Jiang W., Gao Y. (2022). Discovery of the thieno[2,3-d]pyrimidine-2,4-dione derivative 21a: A potent and orally bioavailable gonadotropin-releasing hormone receptor antagonist. Eur. J. Med. Chem..

[92] Schally A.V., Block N.L., Rick F.G. (2017). Discovery of LHRH and development of LHRH analogs for prostate cancer treatment. Prostate.

[93] Limonta P., Manea M. (2013). Gonadotropin-releasing hormone receptors as molecular therapeutic targets in prostate cancer: Current options and emerging strategies. Cancer Treat. Rev..

[94] Janáky T., Juhász A., Bajusz S., Csernus V., Srkalovic G., Bokser L., Milovanovic S., Redding T.W., Rékási Z., Nagy A. (1992). Analogues of luteinizing hormone-releasing hormone containing cytotoxic groups. Proc. Natl. Acad. Sci. USA.

[95] Nagy A., Schally A.V., Armatis P., Szepeshazi K., Halmos G., Kovacs M., Zarandi M., Groot K., Miyazaki M., Jungwirth A. (1996). Cytotoxic analogs of luteinizing hormone-releasing hormone containing doxorubicin or 2-pyrrolinodoxorubicin, a derivative 500-1000 times more potent. Proc. Natl. Acad. Sci. USA.

[96] Liu S.V., Tsao-Wei D.D., Xiong S., Groshen S., Dorff T.B., Quinn D.I., Tai Y.C., Engel J., Hawes D., Schally A.V. (2014). Phase I, dose-escalation study of the targeted cytotoxic LHRH analog AEZS-108 in patients with castration- and taxane-resistant prostate cancer. Clin. Cancer Res. An. Off. J. Am. Assoc. Cancer Res..

[97] Yu S.S., Athreya K., Liu S.V., Schally A.V., Tsao-Wei D., Groshen S., Quinn D.I., Dorff T.B., Xiong S., Engel J. (2017). A Phase II Trial of AEZS-108 in Castration- and Taxane-Resistant Prostate Cancer. Clin. Genitourin. Cancer.

[98] Yates C., Sharp S., Jones J., Topps D., Coleman M., Aneja R., Jaynes J., Turner T. (2011). LHRH-conjugated lytic peptides directly target prostate cancer cells. Biochem. Pharmacol..

[99] Yang W.H., Wieczorck M., Allen M.C., Nett T.M. (2003). Cytotoxic activity of gonadotropin-releasing hormone (GnRH)-pokeweed antiviral protein conjugates in cell lines expressing GnRH receptors. Endocrinology.

[100] Simoni M., Gromoll J., Nieschlag E. (1997). The follicle-stimulating hormone receptor: Biochemistry, molecular biology, physiology, and pathophysiology. Endocr. Rev..

[101] Gonzalez-Robayna I.J., Falender A.E., Ochsner S., Firestone G.L., Richards J.S. (2000). Follicle-Stimulating hormone (FSH) stimulates phosphorylation and activation of protein kinase B (PKB/Akt) and serum and glucocorticoid-lnduced kinase (Sgk): Evidence for A kinase-independent signaling by FSH in granulosa cells. Mol. Endocrinol..

[102] Lizneva D., Rahimova A., Kim S.M., Atabiekov I., Javaid S., Alamoush B., Taneja C., Khan A., Sun L., Azziz R. (2019). FSH Beyond Fertility. Front. Endocrinol..

[103] Richards J.S. (2001). New signaling pathways for hormones and cyclic adenosine 3′,5′-monophosphate action in endocrine cells. Mol. Endocrinol..

[104] Bose C.K. (2008). Follicle stimulating hormone receptor in ovarian surface epithelium and epithelial ovarian cancer. Oncol. Res..

[105] Sanchez A.M., Flamini M.I., Russo E., Casarosa E., Pacini S., Petrini M., Genazzani A.R., Simoncini T. (2016). LH and FSH promote migration and invasion properties of a breast cancer cell line through regulatory actions on the actin cytoskeleton. Mol. Cell. Endocrinol..

[106] Ghinea N. (2018). Vascular Endothelial FSH Receptor, a Target of Interest for Cancer Therapy. Endocrinology.

[107] Mariani S., Salvatori L., Basciani S., Arizzi M., Franco G., Petrangeli E., Spera G., Gnessi L. (2006). Expression and cellular localization of follicle-stimulating hormone receptor in normal human prostate, benign prostatic hyperplasia and prostate cancer. J. Urol..

[108] Dirnhofer S., Berger C., Hermann M., Steiner G., Madersbacher S., Berger P. (1998). Coexpression of gonadotropic hormones and their corresponding FSH- and LH/CG-receptors in the human prostate. Prostate.

[109] Mandrekar P.S., Sheth A.R., Doctor V.M., Zaveri J.P., Sheth N.A. (1990). Immunocytochemical localization of follicle stimulating hormone in normal human stomach. Anat. Rec..

[110] Garde S.V., Sheth A.R., Shah M.G., Kulkarni S.A. (1991). Prostate—An extrapituitary source of follicle-stimulating hormone (FSH): Occurrence, localization, and de novo biosynthesis and its hormonal modulation in primates and rodents. Prostate.

[111] Gartrell B.A., Tsao C.K., Galsky M.D. (2013). The follicle-stimulating hormone receptor: A novel target in genitourinary malignancies. Urol. Oncol..

[112] Perales-Puchalt A., Wojtak K., Duperret E.K., Yang X., Slager A.M., Yan J., Muthumani K., Montaner L.J., Weiner D.B. (2019). Engineered DNA Vaccination against Follicle-Stimulating Hormone Receptor Delays Ovarian Cancer Progression in Animal Models. Mol. Ther. J. Am. Soc. Gene Ther..

[113] Dias J.A., Campo B., Weaver B.A., Watts J., Kluetzman K., Thomas R.M., Bonnet B., Mutel V., Poli S.M. (2014). Inhibition of follicle-stimulating hormone-induced preovulatory follicles in rats treated with a nonsteroidal negative allosteric modulator of follicle-stimulating hormone receptor. Biol. Reprod..

[114] Landomiel F., De Pascali F., Raynaud P., Jean-Alphonse F., Yvinec R., Pellissier L.P., Bozon V., Bruneau G., Crépieux P., Poupon A. (2019). Biased Signaling and Allosteric Modulation at the FSHR. Front. Endocrinol..

[115] Ascoli M., Fanelli F., Segaloff D.L. (2002). The lutropin/choriogonadotropin receptor, a 2002 perspective. Endocr. Rev..

[116] Xiong S., Wang Q., Liu S.V., Montgomery R.B., Stanczyk F.Z., Vallone J.G., Merin N.M., Pinski J. (2015). Effects of luteinizing hormone receptor signaling in prostate cancer cells. Prostate.

[117] Reiter E., McNamara M., Closset J., Hennen G. (1995). Expression and functionality of luteinizing hormone/chorionic gonadotropin receptor in the rat prostate. Endocrinology.

[118] Wang W., Chen Z.X., Guo D.Y., Tao Y.X. (2018). Regulation of prostate cancer by hormone-responsive G protein-coupled receptors. Pharmacol. Ther..

[119] Ingles S.A., Liu S.V., Pinski J. (2013). LHRH and LHR genotypes and prostate cancer incidence and survival. Int. J. Mol. Epidemiol. Genet..

[120] Leuschner C., Hansel W. (2005). Targeting breast and prostate cancers through their hormone receptors. Biol. Reprod..

[121] Bodek G., Kowalczyk A., Waclawik A., Huhtaniemi I., Ziecik A.J. (2005). Targeted ablation of prostate carcinoma cells through LH receptor using Hecate-CGbeta conjugate: Functional characteristic and molecular mechanism of cell death pathway. Exp. Biol. Med..

[122] Hansel W., Leuschner C., Enright F. (2007). Conjugates of lytic peptides and LHRH or betaCG target and cause necrosis of prostate cancers and metastases. Mol. Cell. Endocrinol..

[123] Hansel W., Leuschner C., Gawrońska B., Enright F. (2001). Targeted destruction of prostate cancer cells and xenografts by lytic peptide-betaLH conjugates. Reprod. Biol..

[124] Ciaramella V., Della Corte C.M., Ciardiello F., Morgillo F. (2018). Kisspeptin and Cancer: Molecular Interaction, Biological Functions, and Future Perspectives. Front. Endocrinol..

[125] Ji K., Ye L., Mason M.D., Jiang W.G. (2013). The Kiss-1/Kiss-1R complex as a negative regulator of cell motility and cancer metastasis (Review). Int. J. Mol. Med..

[126] Xoxakos I., Petraki C., Msaouel P., Armakolas A., Grigorakis A., Stefanakis S., Koutsilieris M. (2020). Expression of Kisspeptin (KISS1) and its Receptor GPR54 (KISS1R) in Prostate Cancer. Anticancer Res..

[127] Uenoyama Y., Inoue N., Nakamura S., Tsukamura H. (2019). Central Mechanism Controlling Pubertal Onset in Mammals: A Triggering Role of Kisspeptin. Front. Endocrinol..

[128] Seminara S.B., Messager S., Chatzidaki E.E., Thresher R.R., Acierno J.S., Shagoury J.K., Bo-Abbas Y., Kuohung W., Schwinof K.M., Hendrick A.G. (2003). The GPR54 gene as a regulator of puberty. N. Engl. J. Med..

[129] Martínez-Fuentes A.J., Molina M., Vázquez-Martínez R., Gahete M.D., Jiménez-Reina L., Moreno-Fernández J., Benito-López P., Quintero A., de la Riva A., Diéguez C. (2011). Expression of functional KISS1 and KISS1R system is altered in human pituitary adenomas: Evidence for apoptotic action of kisspeptin-10. Eur. J. Endocrinol..

[130] Kostakis I.D., Agrogiannis G., Vaiopoulos A.G., Mylona E., Patsouris E., Kouraklis G., Koutsilieris M. (2015). A clinicopathological analysis of KISS1 and KISS1R expression in colorectal cancer. APMIS Acta Pathol. Microbiol. Immunol. Scand..

[131] Masui T., Doi R., Mori T., Toyoda E., Koizumi M., Kami K., Ito D., Peiper S.C., Broach J.R., Oishi S. (2004). Metastin and its variant forms suppress migration of pancreatic cancer cells. Biochem. Biophys. Res. Commun..

[132] Curtis A.E., Murphy K.G., Chaudhri O.B., Ramachandran R., Young A.M., Waxman J., Nijher G.M., Bewick G.A., Ghatei M.A., Bloom S.R. (2010). Kisspeptin is released from human prostate cancer cell lines but plasma kisspeptin is not elevated in patients with prostate cancer. Oncol. Rep..

[133] Wang H., Jones J., Turner T., He Q.P., Hardy S., Grizzle W.E., Welch D.R., Yates C. (2012). Clinical and biological significance of KISS1 expression in prostate cancer. Am. J. Pathol..

[134] Asami T., Nishizawa N., Matsui H., Nishibori K., Ishibashi Y., Horikoshi Y., Nakayama M., Matsumoto S., Tarui N., Yamaguchi M. (2013). Design, synthesis, and biological evaluation of novel investigational nonapeptide KISS1R agonists with testosterone-suppressive activity. J. Med. Chem..

[135] MacLean D.B., Matsui H., Suri A., Neuwirth R., Colombel M. (2014). Sustained exposure to the investigational Kisspeptin analog, TAK-448, down-regulates testosterone into the castration range in healthy males and in patients with prostate cancer: Results from two phase 1 studies. J. Clin. Endocrinol. Metab..

[136] Thompson E.L., Amber V., Stamp G.W., Patterson M., Curtis A.E., Cooke J.H., Appleby G.F., Dhillo W.S., Ghatei M.A., Bloom S.R. (2009). Kisspeptin-54 at high doses acutely induces testicular degeneration in adult male rats via central mechanisms. Br. J. Pharmacol..

[137] Petersenn S., Rasch A.C., Penshorn M., Beil F.U., Schulte H.M. (2001). Genomic structure and transcriptional regulation of the human growth hormone secretagogue receptor. Endocrinology.

[138] Gahete M.D., Rincón-Fernández D., Villa-Osaba A., Hormaechea-Agulla D., Ibáñez-Costa A., Martínez-Fuentes A.J., Gracia-Navarro F., Castaño J.P., Luque R.M. (2014). Ghrelin gene products, receptors, and GOAT enzyme: Biological and pathophysiological insight. J. Endocrinol..

[139] Tena-Sempere M. (2008). Ghrelin and reproduction: Ghrelin as novel regulator of the gonadotropic axis. Vitam. Horm..

[140] Kojima M., Hosoda H., Date Y., Nakazato M., Matsuo H., Kangawa K. (1999). Ghrelin is a growth-hormone-releasing acylated peptide from stomach. Nature.

[141] Chow K.B., Sun J., Chu K.M., Tai Cheung W., Cheng C.H., Wise H. (2012). The truncated ghrelin receptor polypeptide (GHS-R1b) is localized in the endoplasmic reticulum where it forms heterodimers with ghrelin receptors (GHS-R1a) to attenuate their cell surface expression. Mol. Cell. Endocrinol..

[142] Cassoni P., Ghé C., Marrocco T., Tarabra E., Allia E., Catapano F., Deghenghi R., Ghigo E., Papotti M., Muccioli G. (2004). Expression of ghrelin and biological activity of specific receptors for ghrelin and des-acyl ghrelin in human prostate neoplasms and related cell lines. Eur. J. Endocrinol..

[143] Jeffery P.L., Herington A.C., Chopin L.K. (2002). Expression and action of the growth hormone releasing peptide ghrelin and its receptor in prostate cancer cell lines. J. Endocrinol..

[144] Hormaechea-Agulla D., Gahete M.D., Jiménez-Vacas J.M., Gómez-Gómez E., Ibáñez-Costa A., L-López F., Rivero-Cortés E., Sarmento-Cabral A., Valero-Rosa J., Carrasco-Valiente J. (2017). The oncogenic role of the In1-ghrelin splicing variant in prostate cancer aggressiveness. Mol. Cancer.

[145] Jeffery P.L., Murray R.E., Yeh A.H., McNamara J.F., Duncan R.P., Francis G.D., Herington A.C., Chopin L.K. (2005). Expression and function of the ghrelin axis, including a novel preproghrelin isoform, in human breast cancer tissues and cell lines. Endocr.-Relat. Cancer.

[146] Jiménez-Vacas J.M., Gómez-Gómez E., Montero-Hidalgo A.J., Herrero-Aguayo V., L-López F., Sánchez-Sánchez R., Guler I., Blanca A., Méndez-Vidal M.J., Carrasco J. (2019). Clinical Utility of Ghrelin-O-Acyltransferase (GOAT) Enzyme as a Diagnostic Tool and Potential Therapeutic Target in Prostate Cancer. J. Clin. Med..

[147] Hormaechea-Agulla D., Gómez-Gómez E., Ibáñez-Costa A., Carrasco-Valiente J., Rivero-Cortés E., L-López F., Pedraza-Arevalo S., Valero-Rosa J., Sánchez-Sánchez R., Ortega-Salas R. (2016). Ghrelin O-acyltransferase (GOAT) enzyme is overexpressed in prostate cancer, and its levels are associated with patient’s metabolic status: Potential value as a non-invasive biomarker. Cancer Lett..

[148] Jiménez-Vacas J.M., Montero-Hidalgo A.J., Gómez-Gómez E., Fuentes-Fayos A.C., Ruiz-Pino F., Guler I., Camargo A., Anglada F.J., Carrasco-Valiente J., Tena-Sempere M. (2021). In1-Ghrelin Splicing Variant as a Key Element in the Pathophysiological Association Between Obesity and Prostate Cancer. J. Clin. Endocrinol. Metab..

[149] Lahlou H., Guillermet J., Hortala M., Vernejoul F., Pyronnet S., Bousquet C., Susini C. (2004). Molecular signaling of somatostatin receptors. Ann. N. Y. Acad. Sci..

[150] Günther T., Tulipano G., Dournaud P., Bousquet C., Csaba Z., Kreienkamp H.J., Lupp A., Korbonits M., Castaño J.P., Wester H.J. (2018). International Union of Basic and Clinical Pharmacology. CV. Somatostatin Receptors: Structure, Function, Ligands, and New Nomenclature. Pharmacol. Rev..

[151] Hasskarl J., Kaufmann M., Schmid H.A. (2011). Somatostatin receptors in non-neuroendocrine malignancies: The potential role of somatostatin analogs in solid tumors. Future Oncol..

[152] Gomes-Porras M., Cárdenas-Salas J., Álvarez-Escolá C. (2020). Somatostatin Analogs in Clinical Practice: A Review. Int. J. Mol. Sci..

[153] Heron I., Thomas F., Dero M., Gancel A., Ruiz J.M., Schatz B., Kuhn J.M. (1993). Pharmacokinetics and efficacy of a long-acting formulation of the new somatostatin analog BIM 23014 in patients with acromegaly. J. Clin. Endocrinol. Metab..

[154] Bauer W., Briner U., Doepfner W., Haller R., Huguenin R., Marbach P., Petcher T.J., Pless J. (1982). SMS 201-995: A very potent and selective octapeptide analogue of somatostatin with prolonged action. Life Sci..

[155] Weckbecker G., Briner U., Lewis I., Bruns C. (2002). SOM230: A new somatostatin peptidomimetic with potent inhibitory effects on the growth hormone/insulin-like growth factor-I axis in rats, primates, and dogs. Endocrinology.

[156] Kosari F., Munz J.M., Savci-Heijink C.D., Spiro C., Klee E.W., Kube D.M., Tillmans L., Slezak J., Karnes R.J., Cheville J.C. (2008). Identification of prognostic biomarkers for prostate cancer. Clin. Cancer Res. An. Off. J. Am. Assoc. Cancer Res..

[157] Sinisi A.A., Bellastella A., Prezioso D., Nicchio M.R., Lotti T., Salvatore M., Pasquali D. (1997). Different expression patterns of somatostatin receptor subtypes in cultured epithelial cells from human normal prostate and prostate cancer. J. Clin. Endocrinol. Metab..

[158] Reubi J.C., Waser B., Schaer J.C., Markwalder R. (1995). Somatostatin receptors in human prostate and prostate cancer. J. Clin. Endocrinol. Metab..

[159] Pedraza-Arévalo S., Hormaechea-Agulla D., Gómez-Gómez E., Requena M.J., Selth L.A., Gahete M.D., Castaño J.P., Luque R.M. (2017). Somatostatin receptor subtype 1 as a potential diagnostic marker and therapeutic target in prostate cancer. Prostate.

[160] Hennigs J.K., Müller J., Adam M., Spin J.M., Riedel E., Graefen M., Bokemeyer C., Sauter G., Huland H., Schlomm T. (2014). Loss of somatostatin receptor subtype 2 in prostate cancer is linked to an aggressive cancer phenotype, high tumor cell proliferation and predicts early metastatic and biochemical relapse. PLoS ONE.

[161] Friedlander T.W., Weinberg V.K., Small E.J., Sharib J., Harzstark A.L., Lin A.M., Fong L., Ryan C.J. (2012). Effect of the somatostatin analog octreotide acetate on circulating insulin-like growth factor-1 and related peptides in patients with non-metastatic castration-resistant prostate cancer: Results of a phase II study. Urol. Oncol..

[162] Karaca B., Degirmenci M., Ozveren A., Atmaca H., Bozkurt E., Karabulut B., Sanli U.A., Uslu R. (2015). Docetaxel in combination with octreotide shows synergistic apoptotic effect by increasing SSTR2 and SSTR5 expression levels in prostate and breast cancer cell lines. Cancer Chemother. Pharmacol..

[163] Mazzucchelli R., Morichetti D., Scarpelli M., Bono A.V., Lopez-Beltran A., Cheng L., Kirkali Z., Montironi R. (2011). Somatostatin receptor subtypes in hormone-refractory (castration-resistant) prostatic carcinoma. Asian J. Androl..

[164] Werner C., Dirsch O., Dahmen U., Grimm M.-O., Schulz S., Lupp A. (2020). Evaluation of Somatostatin and CXCR4 Receptor Expression in a Large Set of Prostate Cancer Samples Using Tissue Microarrays and Well-Characterized Monoclonal Antibodies. Transl. Oncol..

[165] Lupp A., Hunder A., Petrich A., Nagel F., Doll C., Schulz S. (2011). Reassessment of sst(5) somatostatin receptor expression in normal and neoplastic human tissues using the novel rabbit monoclonal antibody UMB-4. Neuroendocrinology.

[166] Ruscica M., Arvigo M., Gatto F., Dozio E., Feltrin D., Culler M.D., Minuto F., Motta M., Ferone D., Magni P. (2010). Regulation of prostate cancer cell proliferation by somatostatin receptor activation. Mol. Cell. Endocrinol..

[167] Durán-Prado M., Gahete M.D., Martínez-Fuentes A.J., Luque R.M., Quintero A., Webb S.M., Benito-López P., Leal A., Schulz S., Gracia-Navarro F. (2009). Identification and characterization of two novel truncated but functional isoforms of the somatostatin receptor subtype 5 differentially present in pituitary tumors. J. Clin. Endocrinol. Metab..

[168] Hormaechea-Agulla D., Jiménez-Vacas J.M., Gómez-Gómez E., L-López F., Carrasco-Valiente J., Valero-Rosa J., Moreno M.M., Sánchez-Sánchez R., Ortega-Salas R., Gracia-Navarro F. (2017). The oncogenic role of the spliced somatostatin receptor sst5TMD4 variant in prostate cancer. FASEB J. Off. Publ. Fed. Am. Soc. Exp. Biol..

[169] Sharma U., Sahu A., Shekhar H., Sharma B., Haque S., Kaur D., Tuli H.S., Mishra A., Ahmad F. (2025). The heat of the battle: Inflammation’s role in prostate cancer development and inflammation-targeted therapies. Discov. Oncol..

[170] Rot A., von Andrian U.H. (2004). Chemokines in innate and adaptive host defense: Basic chemokinese grammar for immune cells. Annu. Rev. Immunol..

[171] Griffith J.W., Sokol C.L., Luster A.D. (2014). Chemokines and chemokine receptors: Positioning cells for host defense and immunity. Annu. Rev. Immunol..

[172] Allen S.J., Crown S.E., Handel T.M. (2007). Chemokine: Receptor structure, interactions, and antagonism. Annu. Rev. Immunol..

[173] Salazar N., Castellan M., Shirodkar S.S., Lokeshwar B.L. (2013). Chemokines and chemokine receptors as promoters of prostate cancer growth and progression. Crit. Rev. Eukaryot. Gene Expr..

[174] Mughees M., Kaushal J.B., Sharma G., Wajid S., Batra S.K., Siddiqui J.A. (2022). Chemokines and cytokines: Axis and allies in prostate cancer pathogenesis. Semin. Cancer Biol..

[175] Hughes C.E., Nibbs R.J.B. (2018). A guide to chemokines and their receptors. FEBS J..

[176] Wang J., Qu C., Xiao P., Liu S., Sun J.-P., Ping Y.-Q. (2025). Progress in structure-based drug development targeting chemokine receptors. Front. Pharmacol..

[177] Murphy C., McGurk M., Pettigrew J., Santinelli A., Mazzucchelli R., Johnston P.G., Montironi R., Waugh D.J.J. (2005). Nonapical and Cytoplasmic Expression of Interleukin-8, CXCR1, and CXCR2 Correlates with Cell Proliferation and Microvessel Density in Prostate Cancer. Clin. Cancer Res..

[178] McClelland S., Maxwell P.J., Branco C., Barry S.T., Eberlein C., LaBonte M.J. (2024). Targeting IL-8 and Its Receptors in Prostate Cancer: Inflammation, Stress Response, and Treatment Resistance. Cancers.

[179] Inoue K., Slaton J.W., Eve B.Y., Kim S.J., Perrotte P., Balbay M.D., Yano S., Bar-Eli M., Radinsky R., Pettaway C.A. (2000). Interleukin 8 Expression Regulates Tumorigenicity and Metastases in Androgen-independent Prostate Cancer. Clin. Cancer Res..

[180] Lehrer S., Diamond E.J., Mamkine B., Stone N.N., Stock R.G. (2004). Serum interleukin-8 is elevated in men with prostate cancer and bone metastases. Technol. Cancer Res. Treat..

[181] Liu X., Peng J., Sun W., Yang S., Deng G., Li F., Cheng J.-W., Gordon J.R. (2012). G31P, an Antagonist against CXC Chemokine Receptors 1 and 2, Inhibits Growth of Human Prostate Cancer Cells in Nude Mice. Tohoku J. Exp. Med..

[182] Yang Y., Li J., Lei W., Wang H., Ni Y., Liu Y., Yan H., Tian Y., Wang Z., Yang Z. (2023). CXCL12-CXCR4/CXCR7 Axis in Cancer: From Mechanisms to Clinical Applications. Int. J. Biol. Sci..

[183] Jang Y.G., Go R.E., Hwang K.A., Choi K.C. (2019). Resveratrol inhibits DHT-induced progression of prostate cancer cell line through interfering with the AR and CXCR4 pathway. J. Steroid Biochem. Mol. Biol..

[184] Zhu W.B., Zhao Z.F., Zhou X. (2019). AMD3100 inhibits epithelial-mesenchymal transition, cell invasion, and metastasis in the liver and the lung through blocking the SDF-1α/CXCR4 signaling pathway in prostate cancer. J. Cell. Physiol..

[185] Reynders N., Abboud D., Baragli A., Noman M.Z., Rogister B., Niclou S.P., Heveker N., Janji B., Hanson J., Szpakowska M. (2019). The Distinct Roles of CXCR3 Variants and Their Ligands in the Tumor Microenvironment. Cells.

[186] Wu Q., Dhir R., Wells A. (2012). Altered CXCR3 isoform expression regulates prostate cancer cell migration and invasion. Mol. Cancer.

[187] Luo Y., Li Q., Yang X., Wei D., Feng B., Li M., Han Y., Zhao J., Lin Y., Li Q. (2021). Overexpression of CXCR7 is a Novel Indicator for Enzalutamide Resistance in Castration-Resistant Prostate Cancer Patients. Dis. Markers.

[188] Bargão Santos P., Lobo J., Félix A., Silva F., Manso R.T., Costa J.O., Lourenço B., Sequeira J.P., Jerónimo C., Patel H.H.R. (2020). The inflammation-related biomarker CXCR7 independently predicts patient outcome after radical prostatectomy. Urol. Oncol..

[189] Mir H., Kapur N., Singh R., Sonpavde G., Lillard J.W., Singh S. (2016). Andrographolide inhibits prostate cancer by targeting cell cycle regulators, CXCR3 and CXCR7 chemokine receptors. Cell Cycle.

[190] Luo Y., Azad A.K., Karanika S., Basourakos S.P., Zuo X., Wang J., Yang L., Yang G., Korentzelos D., Yin J. (2018). Enzalutamide and CXCR7 inhibitor combination treatment suppresses cell growth and angiogenic signaling in castration-resistant prostate cancer models. Int. J. Cancer.

[191] Guo C., Sharp A., Gurel B., Crespo M., Figueiredo I., Jain S., Vogl U., Rekowski J., Rouhifard M., Gallagher L. (2023). Targeting myeloid chemotaxis to reverse prostate cancer therapy resistance. Nature.

[192] Cui L.Y., Chu S.F., Chen N.H. (2020). The role of chemokines and chemokine receptors in multiple sclerosis. Int. Immunopharmacol..

[193] Pang Y., Li H., Gong Y., Jing S., Peng C., Liu W., Zhao Y., Wang H., Kaushik D., Rodriguez R. (2019). Association of CCL2, CCR2 and CCL5 genetic polymorphisms with the development and progression of benign prostatic hyperplasia. Oncol. Rep..

[194] Izumi K., Mizokami A. (2019). Suppressive Role of Androgen/Androgen Receptor Signaling via Chemokines on Prostate Cancer Cells. J. Clin. Med..

[195] Zhao R., Bei X., Yang B., Wang X., Jiang C., Shi F., Wang X., Zhu Y., Jing Y., Han B. (2018). Endothelial cells promote metastasis of prostate cancer by enhancing autophagy. J. Exp. Clin. Cancer Res. CR.

[196] Sicoli D., Jiao X., Ju X., Velasco-Velazquez M., Ertel A., Addya S., Li Z., Andò S., Fatatis A., Paudyal B. (2014). CCR5 receptor antagonists block metastasis to bone of v-Src oncogene-transformed metastatic prostate cancer cell lines. Cancer Res..

[197] Arnatt C.K., Zaidi S.A., Zhang Z., Li G., Richardson A.C., Ware J.L., Zhang Y. (2013). Design, syntheses, and characterization of pharmacophore based chemokine receptor CCR5 antagonists as anti prostate cancer agents. Eur. J. Med. Chem..

[198] Ma J., Wu Y., Wu S., Fang Z., Chen L., Jiang J., Zheng X. (2024). CX3CR1(+)CD8(+) T cells: Key players in antitumor immunity. Cancer Sci..

[199] Lu M., Zhao W., Han S., Lin X., Xu T., Tan Q., Wang M., Yi C., Chu X., Yang W. (2022). Activation of the human chemokine receptor CX3CR1 regulated by cholesterol. Sci. Adv..

[200] Jamieson W.L., Shimizu S., D’Ambrosio J.A., Meucci O., Fatatis A. (2008). CX3CR1 is expressed by prostate epithelial cells and androgens regulate the levels of CX3CL1/fractalkine in the bone marrow: Potential role in prostate cancer bone tropism. Cancer Res..

[201] Liu W., Bian C., Liang Y., Jiang L., Qian C., Dong J. (2017). CX3CL1: A potential chemokine widely involved in the process spinal metastases. Oncotarget.

[202] Liu P., Liang Y., Jiang L., Wang H., Wang S., Dong J. (2018). CX3CL1/fractalkine enhances prostate cancer spinal metastasis by activating the Src/FAK pathway. Int. J. Oncol..

[203] Jiang Y., Guo Y., Han L., Wang S., Wang F., Zhao Y., Wang J., Han J., Liu M., Liu Z. (2026). CX3CL1-CX3CR1 signaling orchestrates malignant progression of prostate cancer through luminal progenitor-macrophage crosstalk. Cell. Oncol..

[204] Xu S., Liu Q., Wang J., Zhang Z., Wang Y., Li L., Zhang T., Fan Y. (2025). CX3CR1 Functions as a Biomarker Associated with Pathological Tumor Staging in the Diagnosis and Prognosis of Prostate Cancer. Iran. J. Immunol. IJI.

[205] Xu C., Wang Y., Ni H., Yao M., Cheng L., Lin X. (2024). The role of orphan G protein-coupled receptors in pain. Heliyon.

[206] Keifi Bajestani A., Alavi M.S., Etemad L., Roohbakhsh A. (2024). Role of orphan G-protein coupled receptors in tissue ischemia: A comprehensive review. Eur. J. Pharmacol..

[207] Zhao M., Wang Z., Yang M., Ding Y., Zhao M., Wu H., Zhang Y., Lu Q. (2021). The Roles of Orphan G Protein-Coupled Receptors in Autoimmune Diseases. Clin. Rev. Allergy Immunol..

[208] Roy N., Parhar I. (2022). Habenula orphan G-protein coupled receptors in the pathophysiology of fear and anxiety. Neurosci. Biobehav. Rev..

[209] Lu Y., Hatzipantelis C.J., Langmead C.J., Stewart G.D. (2024). Molecular insights into orphan G protein-coupled receptors relevant to schizophrenia. Br. J. Pharmacol..

[210] Sakellakis M. (2022). Orphan receptors in prostate cancer. Prostate.

[211] Laschet C., Dupuis N., Hanson J. (2018). The G protein-coupled receptors deorphanization landscape. Biochem. Pharmacol..

[212] Maßberg D., Jovancevic N., Offermann A., Simon A., Baniahmad A., Perner S., Pungsrinont T., Luko K., Philippou S., Ubrig B. (2016). The activation of OR51E1 causes growth suppression of human prostate cancer cells. Oncotarget.

[213] Thomsen M.T., Busk M., Zhang D., Chiu C.L., Zhao H., Garcia-Marques F.J., Bermudez A., Pitteri S., Borre M., Brooks J.D. (2025). The olfactory receptor OR51E2 regulates prostate cancer aggressiveness and modulates STAT3 in prostate cancer cells and in xenograft tumors. BMC Cancer.

[214] Weigle B., Fuessel S., Ebner R., Temme A., Schmitz M., Schwind S., Kiessling A., Rieger M.A., Meye A., Bachmann M. (2004). D-GPCR: A novel putative G protein-coupled receptor overexpressed in prostate cancer and prostate. Biochem. Biophys. Res. Commun..

[215] Fuessel S., Weigle B., Schmidt U., Baretton G., Koch R., Bachmann M., Rieber E.P., Wirth M.P., Meye A. (2006). Transcript quantification of Dresden G protein-coupled receptor (D-GPCR) in primary prostate cancer tissue pairs. Cancer Lett..

[216] Xu L.L., Stackhouse B.G., Florence K., Zhang W., Shanmugam N., Sesterhenn I.A., Zou Z., Srikantan V., Augustus M., Roschke V. (2000). PSGR, a novel prostate-specific gene with homology to a G protein-coupled receptor, is overexpressed in prostate cancer. Cancer Res..

[217] Kim K., Lee J., Choi C., Bae J., Choi H.J., Huh W.K. (2026). Sphingosine-1-phosphate receptor 1 enhances olfactory receptor 51E1-mediated inhibition of proliferation via Src/JNK signaling in prostate cancer cells. Oncol. Rep..

[218] Lin J., Li Q., Lei X., Zhao H. (2022). The emerging roles of GPR158 in the regulation of the endocrine system. Front. Cell Dev. Biol..

[219] Yosten G.L., Harada C.M., Haddock C., Giancotti L.A., Kolar G.R., Patel R., Guo C., Chen Z., Zhang J., Doyle T.M. (2020). GPR160 de-orphanization reveals critical roles in neuropathic pain in rodents. J. Clin. Investig..

[220] Balenga N.A., Henstridge C.M., Kargl J., Waldhoer M. (2011). Pharmacology, signaling and physiological relevance of the G protein-coupled receptor 55. Adv. Pharmacol..

[221] Henstridge C.M., Balenga N.A.B., Kargl J., Andradas C., Brown A.J., Irving A., Sanchez C., Waldhoer M. (2011). Minireview: Recent Developments in the Physiology and Pathology of the Lysophosphatidylinositol-Sensitive Receptor GPR55. Mol. Endocrinol..

[222] Wellendorph P., Bräuner-Osborne H. (2009). Molecular basis for amino acid sensing by family C G-protein-coupled receptors. Br. J. Pharmacol..

[223] Lala T., Hall R.A. (2022). Adhesion G protein-coupled receptors: Structure, signaling, physiology, and pathophysiology. Physiol. Rev..

[224] Azimzadeh P., Talamantez-Lyburn S.C., Chang K.T., Inoue A., Balenga N. (2019). Spatial regulation of GPR64/ADGRG2 signaling by β-arrestins and GPCR kinases. Ann. N. Y. Acad. Sci..

[225] Richter G.H., Fasan A., Hauer K., Grunewald T.G., Berns C., Rössler S., Naumann I., Staege M.S., Fulda S., Esposito I. (2013). G-Protein coupled receptor 64 promotes invasiveness and metastasis in Ewing sarcomas through PGF and MMP1. J. Pathol..

[226] Beaulieu J.M., Gainetdinov R.R. (2011). The physiology, signaling, and pharmacology of dopamine receptors. Pharmacol. Rev..

[227] Peters M.A., Walenkamp A.M., Kema I.P., Meijer C., de Vries E.G., Oosting S.F. (2014). Dopamine and serotonin regulate tumor behavior by affecting angiogenesis. Drug Resist. Updat. Rev. Comment. Antimicrob. Anticancer Chemother..

[228] Wang X., Wang Z.B., Luo C., Mao X.Y., Li X., Yin J.Y., Zhang W., Zhou H.H., Liu Z.Q. (2019). The Prospective Value of Dopamine Receptors on Bio-Behavior of Tumor. J. Cancer.

[229] Samson W.K., Zhang J.V., Avsian-Kretchmer O., Cui K., Yosten G.L., Klein C., Lyu R.M., Wang Y.X., Chen X.Q., Yang J. (2008). Neuronostatin encoded by the somatostatin gene regulates neuronal, cardiovascular, and metabolic functions. J. Biol. Chem..

[230] Zhao J., Wang X., Li T., Chen J., Zhu H., Yang L., Liang Y. (2026). GPR107-EGFR crosstalk promotes ovarian cancer metastasis by regulating IL-6 release via PI3K/AKT/NF-κB signaling pathway. Transl. Oncol..

[231] Liao K., Wang J., Chen J., Zang D., Zhou T., Altankhuyag N., Puseletso M., Wang J., Cai C., Zhou F. (2026). GPR107 promotes autophagy secretion in psoriatic keratinocytes by inhibiting BECN1 K48-linked ubiquitination. Cell Death Dis..

[232] Singh A.K., Lin H.H. (2021). The role of GPR56/ADGRG1 in health and disease. Biomed. J..

[233] Jiang X., Xu X., Wu M., Guan Z., Su X., Chen S., Wang H., Teng L. (2018). GPRC5A: An Emerging Biomarker in Human Cancer. BioMed Res. Int..

[234] Wiley S.Z., Sriram K., Salmerón C., Insel P.A. (2019). GPR68: An Emerging Drug Target in Cancer. Int. J. Mol. Sci..

[235] Filipowska J., Kondegowda N.G., Leon-Rivera N., Dhawan S., Vasavada R.C. (2022). LGR4, a G Protein-Coupled Receptor with a Systemic Role: From Development to Metabolic Regulation. Front. Endocrinol..

[236] Mariana S.-M., Brenda R.-P., Heriberto P.-G., Cristina L., David B., Guadalupe Á.-L. (2025). GPER1 activation by estrogenic compounds in the inflammatory profile of breast cancer cells. J. Steroid Biochem. Mol. Biol..

[237] Yao Y., Gong Y., Cao K. (2026). Potential Roles of G Protein-Coupled Receptor 30 (GPR30) in Migraine Pathophysiology. J. Pain Res..

[238] Qian H., Xuan J., Liu Y., Shi G. (2016). Function of G-Protein-Coupled Estrogen Receptor-1 in Reproductive System Tumors. J. Immunol. Res..

[239] Huang B.X., Hu X., Kwon H.-S., Fu C., Lee J.-W., Southall N., Marugan J., Kim H.-Y. (2020). Synaptamide activates the adhesion GPCR GPR110 (ADGRF1) through GAIN domain binding. Commun. Biol..

[240] Lee J.-W., Huang B.X., Kwon H., Rashid M.A., Kharebava G., Desai A., Patnaik S., Marugan J., Kim H.-Y. (2016). Orphan GPR110 (ADGRF1) targeted by N-docosahexaenoylethanolamine in development of neurons and cognitive function. Nat. Commun..

[241] Tian J.-Y., Chi C.-L., Bian G., Guo F.-J., Wang X.-Q., Yu B. (2021). A novel GPCR target in correlation with androgen deprivation therapy for prostate cancer drug discovery. Basic Clin. Pharmacol. Toxicol..

[242] Schmidt K.T., Huitema A.D.R., Chau C.H., Figg W.D. (2021). Resistance to second-generation androgen receptor antagonists in prostate cancer. Nat. Rev. Urol..

[243] Waldron N.R., Silva D., Westaby D., Jiménez-Vacas J., Taylor J., Sharp A. (2026). Decoding the Raf-Mek-Erk-Rsk pathway in prostate cancer: From molecular mechanisms to clinical opportunities. Br. J. Cancer.

[244] Lin H.K., Hu Y.C., Yang L., Altuwaijri S., Chen Y.T., Kang H.Y., Chang C. (2003). Suppression versus induction of androgen receptor functions by the phosphatidylinositol 3-kinase/Akt pathway in prostate cancer LNCaP cells with different passage numbers. J. Biol. Chem..

[245] Yan Y., Huang H., Dehm S.M., Tindall D.J. (2019). Interplay Among PI3K/AKT, PTEN/FOXO and AR Signaling in Prostate Cancer. Prostate Cancer: Cellular and Genetic Mechanisms of Disease Development and Progression.

[246] Zhao Y., Tindall D.J., Huang H. (2014). Modulation of androgen receptor by FOXA1 and FOXO1 factors in prostate cancer. Int. J. Biol. Sci..

[247] Lin H.K., Yeh S., Kang H.Y., Chang C. (2001). Akt suppresses androgen-induced apoptosis by phosphorylating and inhibiting androgen receptor. Proc. Natl. Acad. Sci. USA.

[248] Quistini A., Chierigo F., Fallara G., Depalma M., Tozzi M., Maggi M., Jannello L.M.I., Pellegrino F., Mantica G., Terracciano D. (2025). Androgen Receptor Signalling in Prostate Cancer: Mechanisms of Resistance to Endocrine Therapies. Res. Rep. Urol..

[249] Aurilio G., Cimadamore A., Mazzucchelli R., Lopez-Beltran A., Verri E., Scarpelli M., Massari F., Cheng L., Santoni M., Montironi R. (2020). Androgen Receptor Signaling Pathway in Prostate Cancer: From Genetics to Clinical Applications. Cells.

[250] Case T.C., Merkel A., Ramirez-Solano M., Liu Q., Sterling J.A., Jin R. (2021). Blocking GRP/GRP-R signaling decreases expression of androgen receptor splice variants and inhibits tumor growth in castration-resistant prostate cancer. Transl. Oncol..

[251] Zhu X., Farsh T., Vis D., Yu I., Li H., Liu T., Sjöström M., Shrestha R., Kneppers J., Severson T. (2024). Genomic and transcriptomic features of androgen receptor signaling inhibitor resistance in metastatic castration-resistant prostate cancer. J. Clin. Investig..

[252] Kwon W.A., Song Y.S., Lee M.K. (2024). Strategic Advances in Combination Therapy for Metastatic Castration-Sensitive Prostate Cancer: Current Insights and Future Perspectives. Cancers.

[253] Armstrong A.J., Geva R., Chung H.C., Lemech C., Miller W.H., Hansen A.R., Lee J.S., Tsai F., Solomon B.J., Kim T.M. (2024). CXCR2 antagonist navarixin in combination with pembrolizumab in select advanced solid tumors: A phase 2 randomized trial. Investig. New Drugs.

[254] Gleicher S., Porter B.A., Nath D., Li G., Khanna R., Goldberg H., Kortylewski M., Bratslavsky G., Kotula L. (2021). Novel Target Opportunities in Non-Metastatic Castrate Resistant Prostate Cancer. Cancers.

[255] Thakur M.K., Heilbrun L., Dobson K., Boerner J., Stark K., Li J., Smith D., Heath E., Fontana J., Vaishampayan U. (2018). Phase I Trial of the Combination of Docetaxel, Prednisone, and Pasireotide in Metastatic Castrate-Resistant Prostate Cancer. Clin. Genitourin. Cancer.

[256] Li S., Fong K.W., Gritsina G., Zhang A., Zhao J.C., Kim J., Sharp A., Yuan W., Aversa C., Yang X.J. (2019). Activation of MAPK Signaling by CXCR7 Leads to Enzalutamide Resistance in Prostate Cancer. Cancer Res..

[257] Rafiei S., Gui B., Wu J., Liu X.S., Kibel A.S., Jia L. (2019). Targeting the MIF/CXCR7/AKT Signaling Pathway in Castration-Resistant Prostate Cancer. Mol. Cancer Res. MCR.

[258] Paudyal P., Xie Q., Vaddi P.K., Henry M.D., Chen S. (2017). Inhibiting G protein βγ signaling blocks prostate cancer progression and enhances the efficacy of paclitaxel. Oncotarget.

[259] Kübler E., Albrecht H. (2018). Large set data mining reveals overexpressed GPCRs in prostate and breast cancer: Potential for active targeting with engineered anti-cancer nanomedicines. Oncotarget.

[260] Li S., Chen J., Chen X., Yu J., Guo Y., Li M., Pu X. (2022). Therapeutic and prognostic potential of GPCRs in prostate cancer from multi-omics landscape. Front. Pharmacol..

[261] Rajagopal S., Shenoy S.K. (2018). GPCR desensitization: Acute and prolonged phases. Cell. Signal..

[262] Congreve M., de Graaf C., Swain N.A., Tate C.G. (2020). Impact of GPCR Structures on Drug Discovery. Cell.

[263] Addis P., Bali U., Baron F., Campbell A., Harborne S., Jagger L., Milne G., Pearce M., Rosethorne E.M., Satchell R. (2024). Key aspects of modern GPCR drug discovery. SLAS Discov..

[264] High P., Carmon K.S. (2023). G protein-coupled receptor-targeting antibody-drug conjugates: Current status and future directions. Cancer Lett..

[265] Sriram K., Moyung K., Corriden R., Carter H., Insel P.A. (2019). GPCRs show widespread differential mRNA expression and frequent mutation and copy number variation in solid tumors. PLoS Biol..

[266] de Bono J.S., Guo C., Gurel B., De Marzo A.M., Sfanos K.S., Mani R.S., Gil J., Drake C.G., Alimonti A. (2020). Prostate carcinogenesis: Inflammatory storms. Nat. Rev. Cancer.

[267] Heidegger I., Fotakis G., Offermann A., Goveia J., Daum S., Salcher S., Noureen A., Timmer-Bosscha H., Schäfer G., Walenkamp A. (2022). Comprehensive characterization of the prostate tumor microenvironment identifies CXCR4/CXCL12 crosstalk as a novel antiangiogenic therapeutic target in prostate cancer. Mol. Cancer.

[268] Li D., Xu W., Chang Y., Xiao Y., He Y., Ren S. (2023). Advances in landscape and related therapeutic targets of the prostate tumor microenvironment. Acta Biochim. Biophys. Sin..

[269] Sorrentino C., Di Carlo E. (2023). Molecular Targeted Therapies in Metastatic Prostate Cancer: Recent Advances and Future Challenges. Cancers.

[270] Li M., Kwantwi L.B., Wang C., Xiao Q. (2025). Tumor microenvironment-mediated immune evasion and resistance in prostate cancer: Mechanisms, cross-talk, and therapeutic opportunities. Clin. Exp. Med..

[271] Vindrieux D., Escobar P., Lazennec G. (2009). Emerging roles of chemokines in prostate cancer. Endocr.-Relat. Cancer.

